# Dynamics of Simultaneous and Imitative Bodily Coordination in Trust and Distrust

**DOI:** 10.3389/fpsyg.2018.01546

**Published:** 2018-08-28

**Authors:** Carlos Cornejo, Esteban Hurtado, Zamara Cuadros, Alejandra Torres-Araneda, Javiera Paredes, Himmbler Olivares, David Carré, Juan P. Robledo

**Affiliations:** ^1^Laboratorio de Lenguaje Interacción y Fenomenología, Escuela de Psicología, Pontificia Universidad Católica de Chile, Santiago, Chile; ^2^Departamento de Psicología, Universidad de Concepción, Concepción, Chile; ^3^Carrera de Psicología, Universidad Arturo Prat, Iquique, Chile; ^4^Centre for Music and Science, University of Cambridge, Cambridge, United Kingdom

**Keywords:** interpersonal coordination, trust, mocap, anatomical imitation, mirroring, imitation, simultaneous coordination

## Abstract

Body synchronization between interacting people involves coordinative movements in time, space and form. The introduction of newer technologies for automated video analysis and motion tracking has considerably improved the accurate measurement of coordination, particularly in temporal and spatial terms. However, the form of interpersonal coordination has been less explored. In the present study we address this gap by exploring the effect of trust on temporal and morphological patterns of interpersonal coordination. We adapted an optical motion-capture system to record spontaneous body movements in pairs of individuals engaged in natural conversations. We conducted two experiments in which we manipulated trust through a breach of expectancy (Study 1: 10 trustful and 10 distrustful participants) and friendship (Study 2: 20 dyads of friends and 20 dyads of strangers). In Study 1, results show the participants' strong, early mirror-like coordination in response to the confederates' breach of trust. In Study 2, imitative coordination tended to be more pronounced in pairs of friends than in pairs of non-friends. Overall, our results show not only that listeners move in reaction to speakers, but also that speakers react to listeners with a chain of dynamic coordination patterns affected by the immediate disposition of, and long-term relationship with, their interlocutors.

## Introduction

When people interact socially, they tend to spontaneously synchronize their bodily movements in time, space and form (Bernieri et al., 1988; Bernieri and Rosenthal, 1991). This pervasive feature of social exchanges between people is referred to as interpersonal coordination (Lumsden et al., 2012; Rio and Warren, 2016; Good et al., 2017). It occurs continuously during daily-life joint actions, for instance when people walk together (van Ulzen et al., 2008), rock in rocking chairs (Richardson et al., 2005), play sports games (Passos and Chow, 2016), handclap (Néda et al., 2000), dance in a club (Ellamil et al., 2016) and chat (Paxton and Dale, 2013a,b,c), among others.

The most widespread explanation of this phenomenon points to its role in creating and maintaining ties of a social and affective nature (Semin and Cacioppo, 2008; Marsh et al., 2009; Cacioppo et al., 2014). Thus, interpersonal synchrony would favor the emergence of a common ground that enriches affiliation in social relationships (Lumsden et al., 2014). Consequently, some researchers claim that interpersonal coordination satisfies communicative (Scheflen, 1964; Wallbott, 1996; Latif et al., 2014), affiliative (de Waal, 2008, 2009; Lumsden et al., 2014) and social functions (Hatfield et al., 1994; Semin and Cacioppo, 2008; Cacioppo et al., 2014); some even postulate that these functions are part of a biological mechanism underlying the configuration of coordinated movement patterns among congeners (Hatfield et al., 1994; de Waal, 2008, 2009; Semin and Cacioppo, 2008; Latif et al., 2014). Empirical approaches supporting these statements have focused on studying the psychosocial factors that modulate movement coordination when people interact, as well as the social consequences of such coordination (Zhao et al., 2015).

Studies of psychosocial factors include individual and contextual variables that influence interpersonal coordination. Personal features reported to influence interpersonal coordination are social competence (Schmidt et al., 1994), social motives (Lumsden et al., 2012), physical attractiveness (Zhao et al., 2015) and persistent concerns about social evaluations (Varlet et al., 2014). For example, interactions with an attractive virtual agent have been found to improve the stability of interpersonal coordination compared with interactions with a less attractive virtual agent (Zhao et al., 2015). Research has also shown that, relative to a pro-self mindset, a pro-social mindset is associated with a higher degree of interpersonal bodily coordination. Patients with social-cognition deficits and mental disorders exhibit impaired social motor coordination. Those with autism spectrum disorder (Marsh et al., 2013), social anxiety disorder (Varlet et al., 2014) and schizophrenia (Varlet et al., 2012) have been reported to be less able to coordinate with others.

In addition to individual variables, studies of socio-contextual variables have shown that the affective tone inherent in specific interactional contexts can change the ways in which people coordinate with one another. For example, body coordination has been found to increase to a greater extent during non-conflicting interactions than during conflicting interactions (Paxton and Dale, 2013a,b,c; Hammal et al., 2014). Similarly, other studies have revealed that individuals often become spontaneously synchronized when participating in competitive, cooperative or fun interactional contexts (Valdesolo et al., 2010; Rodrigues and Passos, 2013; Tschacher et al., 2014). By contrast, in-phase synchrony is significantly reduced when participants adopt a negative affective tone toward a tardy confederate (Miles et al., 2010). Together, these findings suggest that psychosocial factors modulate the dynamics of interpersonal coordination. Positive factors related to people, their relationships and the social context seem to activate or enhance synchronized co-activity; conversely, negative factors appear to inhibit, or even prevent, coordination.

Studies have also been conducted on the effects of interpersonal coordination on social exchanges. For example, after a period of synchronized activity, variables related to interpersonal affect improve, such as positive affect (Tschacher et al., 2014), trust (Launay et al., 2013), social bonding (Tarr et al., 2016), affiliation (Hove and Risen, 2009), and rapport (Chartrand and Bargh, 1999; Marzoli et al., 2011; Vacharkulksemsuk and Fredrickson, 2012). Some studies have also demonstrated that coordinated movements strengthen several pro-social behaviors. For example, episodes of coordinated activity enhance helpfulness in infants (Cirelli et al., 2014a,b, 2016) and children (Kirschner and Tomasello, 2010; Kirschner and Ilari, 2014; Endedijk et al., 2015). Similar results have been reported for adults. After a period of simultaneous coordination, cooperation (Wiltermuth and Heath, 2009; Kokal et al., 2011), compassion and altruistic behavior (Valdesolo and DeSteno, 2011) improve between adults performing joint tasks.

Although the empirical evidence has provided relevant knowledge about the close and bidirectional relation between body coordination and social-affective ties, ecological validity has not always been addressed. In some cases, experimental designs involve interactional situations that are distant from real social life (Musa et al., 2015; Cornejo et al., 2017a,b). Recent naturalistic and semi-naturalistic research framing coordination in conversational contexts seems to face this challenge. For example, Paxton and Dale (2017) studied natural conversations between people, identifying zero-lag and time-delayed body synchronization patterns in dyadic affiliative conversations. They found that these coordinated patterns increase to a larger degree during affiliative conversations than during argumentative discussions. Latif et al. (2014) also found synchronization in real conversations between pairs of friends and strangers; however, they found that pairs of friends often exhibited greater levels of coordination (in both zero-lag and time-delayed synchronization patterns) than pairs of strangers. In another study of natural conversations, Tschacher et al. (2014) found greater synchrony between couples of strangers who self-reported positive affect than between couples of strangers who self-reported negative affect.

The results of naturalistic studies have replicated and extended previous works analyzing dyadic conversations in real psychotherapy settings. It has been reported that body movement coordination is enhanced between therapists and patients with a high-quality relationship, compared with those involved in a low-quality relationship (Nagaoka et al., 2007; Nagaoka and Komori, 2008). Some researchers have claimed that greater coordination between affiliated interaction partners may be due to the perceived similarity between them (Dunne and Ng, 1994; Feyereisen, 1994; Miles et al., 2011). Other proposed explanations emphasize that common knowledge about conversation topics may produce synchronized patterns of coordination, even in pairs of strangers (Clark, 1996; Keysar et al., 2000; Richardson et al., 2007; Fast et al., 2009).

Findings from naturalistic research have enabled an understanding of interpersonal coordination sensitivity to relational and contextual factors. In particular, such research has garnered valuable knowledge about how high- and low-level conversational constraints impact the temporal-spatial dimensions of interpersonal coordination. This advance has been made possible by the use of automated analyses of video-recorded movements, such as Motion Energy Analysis (Ramseyer and Tschacher, 2011; Tschacher et al., 2014), Frame Differencing Method (Paxton and Dale, 2013a,b,c) and Correlation Map Analysis (Latif et al., 2014). Starting from global video images of interacting partners, automated analyses of video deliver highly accurate temporal information.

Although previous research has examined the temporal-spatial dynamics of synchronized movement, the morphological dynamics of interpersonal coordination in natural settings remains unexplored. Evidence from the developmental study of imitation has revealed two types of time-delayed body coordination–namely, mirror-like and anatomical. Mirror-like coordination comprises coordinated movements that reflect the actions of the interaction partners (Pierpaoli et al., 2014), so that a spatial correspondence can be observed between the imitator's movements and the movements of the model (Chiavarino, 2012). For example, the hearer moves her right arm when the speaker moves her left one. By contrast, anatomical coordination refers to coordinative movements that reconstruct the interactant body-scheme (Pierpaoli et al., 2014), establishing an anatomical correspondence between the imitator's movements and the movements of the model (Chiavarino, 2012). For instance, the hearer moves her right arm when the speaker moves her right one.

To date, no research into interpersonal coordination has been conducted to distinguish between different forms of synchronized movement. To further naturalistic research into interpersonal coordination, we conducted two studies to observe the effects of trust on temporal and morphological patterns of coordinated movements between two people engaged in a real conversation. We emulated experimental procedures described in earlier naturalistic studies (Paxton and Dale, 2013a,b,c, 2017; Latif et al., 2014), but used different methods of capture and analysis. Given the limited utility of automated analysis of video-recordings for the description of the forms of coordination, we used a motion capture system to track participants' interactions.

Although motion capture systems have been previously used to study interpersonal coordination in joint tasks (Fine and Amazeen, 2011; Varlet et al., 2011; Ragert et al., 2013; Vesper et al., 2013; Fine et al., 2015; Gueugnon et al., 2016; Llobera et al., 2016; Preissmann et al., 2016; Chang et al., 2017; Romero et al., 2017), the present research is, to the best of our knowledge, the first to adapt motion capture to record spontaneous body movements of individuals engaged in real conversations. This technique has the advantage of allowing us to record accurate and detailed measurements of the attributes that shape interpersonal coordination, such as time (e.g., zero-lag or time-delayed), space (e.g., amplitude and direction of synchronized motions) and form (e.g., mirror-like and anatomical coordination).

Our interest in trust is twofold. On the one hand, based on different disciplines and theoretical approaches, trust has been considered a fundamental part of social life (Rotter, 1967, 1971, 1980; Luhmann, 1979; Simpson, 2007a,b). Trust plays a major role in establishing links with others: “…[it] is the lubricant that makes a society run smoothly” (de Waal, 2009, 224). This subjective state is understood as the expectation that the other will not deceive you (Rousseau et al., 1998; de Waal, 2009). It comprises “…[it] the intention to accept vulnerability based upon positive expectations of the intentions or behavior of another” (Rousseau et al., 1998, p. 395). Trust implies a set of personal beliefs, affects and positive expectations about others (Lewis and Weigert, 1985; Rempel et al., 1985; Das and Teng, 2001; Inkpen and Currall, 2004). These beliefs, affects and expectations about others determine feelings and behaviors (Kramer and Carnevale, 2001). When positive beliefs and expectations about another are confirmed, feelings and behaviors of proximity emerge, thereby promoting trust (DiYanni and Kelemen, 2008; Ma et al., 2015). Conversely, when positive beliefs and expectations about another are violated, negative feelings and withdrawal behaviors emerge, leading to distrust (Sitkin and Roth, 1993; Maddux et al., 2008).

On the other hand, little is known about the impact of trust on the way that people coordinate with each other during social exchanges. In fact, most studies on trust favor correlational approaches that verify the association between interpersonal coordination and the participants' self-reported trust (Marzoli et al., 2011; Launay et al., 2013). Besides the problems of social desirability inherent to self-reports (Haeffel and Howard, 2010), the lack of proper explanatory models limits the study of the impact of trust and distrust on interactional dynamics. In this article, we focus on trust as an interactional process that can change throughout a conversation and impact the bodily coordination displayed between subjects. We explored this phenomenon by examining real interactions by means of online techniques. Conceptually, we have understand trust as an interactional process that manifests in spontaneous body movements.

## The current study

We conducted two studies that aimed to describe patterns of interpersonal coordination in situations of trust and distrust. In both studies, participants partook in a natural conversation within an experimental setting, where body movements were tracked using an optical motion-capture (or “mocap”) system. We adapted the mocap system to track the body movements of participants engaged in natural conversation. By creating large elastic bands to hold small reflective markers around the body in key positions on the torso and limbs, we were able to minimize the level of interference with participants' movement and clothing, as compared with that observed when subjects were asked to wear special motion-tracking suits. Participants were paired and then made to engage in conversation based on a topic proposed by the researchers.

In Study 1, we manipulated trust and measured its impact on interpersonal coordination. We compared patterns of interpersonal coordination in a trust condition vs. a non-trust condition, in which a confederate produced a breach of trust. In the middle of a normal conversation, a confederate actor confessed to be working at the laboratory. The objective of this manipulation was to trigger distrust toward the confederate by violating the participants' expectation that the confederate was interacting with another naive participant. We recorded and compared the coordination patterns between participants before and during the breach of trust. Since previous research has revealed that negative affective states linked to distrust are associated with decreases in interpersonal coordination, we expected to find more coordination between participants in a trust condition than between those in a non-trust condition. In addition, we expected to find more coordination before the breach of trust than after it. We postulated, therefore, that such difference should not appear in the control condition, where no breach of trust was implemented.

Study 2 compared patterns of interpersonal coordination between contexts in which trust pre-existed (due to friendship between participants–friend condition) and contexts in which trust had to be built from scratch (non-friend condition). Previous findings have shown that people trust more in their friends than in strangers (Glaeser et al., 2000; Binzel and Fehr, 2013). In line with this and the aforementioned finding of Latif et al. (2014), we expected more coordination between friends than between strangers.

## Materials and methods

As detailed in the following sections, we conducted two studies that shared the same physical setup and technical data recording procedures.

### Materials

To record the body movements of two interacting people at the same time, a room was equipped with an optical motion-capture system consisting of 36 purpose specific cameras (Natural Point Prime-41)^1^ wired to a personal computer. The application package supplied by the system manufacturer (Motive optical motion capture software)^2^ was used for calibration, 3D reconstruction, and motion recording at 120 frames per second. Although our equipment is capable of recording motion in any direction in 3D space, our analysis will focus exclusively on the proximal axis (how close or far participants are). It is important to note that this axis is not necessarily parallel to the 2 image axes of any of our cameras^3^. Cameras were set up close to the ceiling, forming a rectangular perimeter above and surrounding the participants. Cameras were located so that the capture area was at least 3 meters x 4 meters x 2 meters (width, depth, height).

The Optitrack system fills a room with infrared light and tracks the position of small infrared reflective spherical markers. It was designed for the production of motion pictures and computer graphics. Therefore, this system is best suited for participants who are willing to go through a skeleton calibration procedure, who can use special suits and who are capable of repeating short takes several times. Under these conditions, the system is capable of adjusting a skeleton model to each actor and automatically tracking body parts.

The conditions of our human interaction study differed from those outlined above, as participants needed to be as comfortable as possible and were not required to wear special suits. In addition, the recordings of participants' movements lasted for several minutes, and they were not required to repeat any part of their movements. Despite these different conditions, an optical motion-capture system was ideal for our study because it allows participants to move freely in a reasonably natural setting.

We modified the recommended recording protocol according to our specific needs. We reduced the number of markers on each person's body from 37 to 15, in order to increase the participants' comfort. Markers were located on the feet (2x), the knees (2x), the lower back (2x), the upper back (2x), the hands (2x), the elbows (2x), and the head (3x). Since the system tracks markers individually, no performance degradation results from reducing the number of markers. Markers were attached using elastic bands, which were adjustable and comfortable. While this increased comfort for participants, it meant that each body part was tracked as a single marker instead of an automatically detectable pattern made from several markers. This scheme forced us to manually label body parts off-line. We wrote scripts to do a 3D visualization of markers, which allowed us to unambiguously indicate a correspondence between markers and body parts. After the experiment, several participants spontaneously told us that they forgot about the markers. In addition, this setup allowed for short startup times. In each session, two participants wore the markers and sat on cube-shaped seats, facing one another. The absence of a chair back allowed the back markers to be easily visible to the cameras, and participants were able to move freely.

David Eagleman's short story “Sum” was used as a topic of conversation^4^. Six questions were printed on cards to guide the conversation: (1) “What's your name? What's your major, and why did you choose it?”; (2) “Did you like the text? Why?”; (3) “What comes to your mind after reading the text?”; (4) “Which part of the story touched you the most? Why?"; (5) “Do you feel that the text reflects your life? How?”; and (6) “Did you remember any event or person?”

### Common procedure

Participants were recruited by invitation at the beginning of class sessions, after approval from their teachers. No teacher was related to this research project. A participant was brought into the laboratory by an assistant, signed an informed consent form, and was asked to read the short story. Another participant was brought into an adjacent room for the exact same procedure. Fifteen mocap markers were attached to participants' bodies using elastic bands. Then, the second participant was brought into the laboratory with markers already attached and was introduced to the participant already there. The researcher asked participants to sit on two cubes facing one another. Directions were given on how to proceed with a conversation during the session. While the mocap system recorded their movements, they would go through each question printed on the cards, talking as long as they wanted to answer them. Each question was to be answered by both participants. They would take turns reading each question and being the first to answer. As a result, one participant would read and begin answering questions 1, 3, and 5, while the other would do the same for questions 2, 4, and 6. This scheme was designed to avoid a clear asymmetry between participants.

### Study 1

Our first study sought to investigate the effect of sudden changes in trust on body coordination patterns. This change was elicited by manipulating the violation of expectancy of naïve participants about another person.

#### Participants

The participants were 20 undergraduate students from Pontificia Universidad Católica de Chile (ages: *M* = 20.55, *SD* = 1.32 years old; 12 females). All participants voluntarily signed up for the experiment, and they were given a lunch voucher for their participation. Ethical approval for this study was given by the Ethical Committee of Social Sciences at the Pontificia Universidad Católica de Chile, following the Declaration of Helsinki. Each participant took part in an experimental session with a same-sex confederate (ages: *M* = 21.50, *SD* = 0.58). Confederates were acting students at the same university, and they were recruited and economically compensated for this study. There were 4 confederates. Each participated from 3 to 8 times. Each participated in both condition. In all cases, the participant and the confederate did not know each other, which was explicitly asked. Participants were randomly assigned either to a trust or non-trust group (of equal size). No participant decided to withdraw from the study. In each session, the confederate was labeled “subject A,” and the participant was labeled “subject B.”

#### Experimental manipulation

In Study 1, the person who was prepared in an adjacent room was a confederate. After entering the experimental setting, she/he was introduced to the actual participant as another participant. To ensure consistent content in the confederate's answers across sessions, she/he had to learn and practice a set of pre-designed answers created by the research team behind the study. In the trust condition, the confederate answered all the questions, acting as a naive participant. In the non-trust condition, the confederate acted as a naive participant until the fourth question. When answering the fifth question, she/he was instructed to reveal that she/he had participated several times in the same experiment and worked regularly with the research team, making sure that the participant understood. This experimental manipulation was designed to disrupt the participant's trust. After going through all questions, the confederate was sent to another room, markers were removed, and the participant was debriefed, which included clarifications about the role of the confederate, the experimental manipulation, and the research motivations.

To assess the experimental manipulation, two condition-blind judges with experience in video analysis and non-verbal behavior were recruited. The two judges were coauthors of this work; neither took part in motion recording sessions. They were asked to rate, on a 1–5 scale, the discomfort perceived in the naive participants during the fifth question (when the manipulation occurred). The 20 videos (10 for each condition) showed only the naive participant's image (excluding the confederate) and were presented without audio, so judges were blind to which experimental condition corresponded to each video. Judges were also asked to take note of the beginning and ending times of the discomfort interaction segment in each recording, except for cases rated as 1, which meant “no-discomfort.” We subtracted 1 from the given discomfort ratings (so that “no-discomfort” became 0) and computed the duration in seconds of the discomfort segments. Additionally, a discomfort score was computed by multiplying the two previous variables, to include both the level of discomfort and the duration in a single metric. We fit one mixed ANOVA model for each of those three response variables, after applying a natural logarithm with the purpose of reducing positive skewness. In each case, the condition from which each video was taken (trust or non-trust) was a between-group factor, and the judge (A or B) was a within-group factor. Our results showed that, on average, discomfort ratings were higher for non-trust (*M* = 2.00) than trust (*M* = 1.05) conditions [*F*_(1, 18)_ = 5.7623, *p* = 0.0274]. Additionally, the observed discomfort segment durations were longer for non-trust (*M* = 27.85 s) than trust (*M* = 13.35 s) conditions [*F*_(1, 18)_ = 6.2353, *p* = 0.0224]. A similar pattern was observed in discomfort scores [non-trust: *M* = 79.50; trust: *M* = 34.65; *F*_(1, 18)_ = 6.2986, *p* = 0.0286]. There were no statistically significant effects of rater [on discomfort ratings: *F*_(1, 18)_ = 0.038, *p* = 0.848; on durations: *F*_(1, 18)_ = 0.716, *p* = 0.409; on discomfort scores: *F*_(1, 18)_ = 0.223, *p* = 0.640] or rater-condition interaction [on discomfort ratings: *F*_(1, 18)_ = 0.177, *p* = 0.679; on durations: *F*_(1, 18)_ = 0.354, *p* = 0.559; on discomfort scores: *F*_(1, 18)_ = 0.290, *p* = 0.597].

As shown in Table 1, durations are longer in the non-trust group when trust was breached, but similar between groups before breach. This is further confirmation that manipulation had an effect.

**Table 1:** Before breach (4th) and breach (5th) question durations by group.

**Question**	**Trust group duration (s)**	**Non-trust group duration (s)**	**|*t*|for difference of log-duration means**	**DF**	***p***
4th	*M* = 376.30	*M* = 368.70	0.45	14.43	0.660
	*SD* = 88.61	*SD* = 145.04			
5th	*M* = 525.50	*M* = 964.50	3.69	17.00	0.002
	*SD* = 88.61	*SD* = 145.04			
**Question**	**Friends group duration (s)**	**Strangers group duration (s)**	|*t*|**for difference of log-duration means**	**DF**	*p*
4th	*M* = 202.95	*M* = 273.95	2.01	37.93	0.052
	*SD* = 78.10	*M* = 131.36			
5th	*M* = 299.05	*M* = 388.80	1.75	34.839	0.149
	*M* = 108.39	*M* = 211.43			

*Actual trust breach manipulation only occurs during 5th question in non-trust group. Welch t-tests are shown for differences in logaritmized durations*.

### Study 2

As people usually trust more in their friends than in others that they do not know, the second study investigated the effect of friendship on body coordination patterns. It included pairs of friends and pairs of non-friends, i.e., people who met for the first time, as participants.

#### Participants

Forty dyads of students from Pontificia Universidad Católica de Chile (mean age=21.39 years old; 41 female) participated in this study. All participants voluntarily signed up for the experiment, and they were given a lunch voucher for their participation. Ethical approval for this study was given by the Ethical Committee of Social Sciences at the Pontificia Universidad Católica de Chile, following the Declaration of Helsinki. No participants decided to withdraw from the study.

#### Experimental manipulation

There were no confederates in this experiment. Experimental manipulation consisted of having two conditions: friends and non-friends. To produce the friends condition, participants who signed up for the study were asked to also invite a friend. Only dyads that reported a friendship of at least 6 months where included. The non-friends condition included 20 dyads of individuals who had not met before.

Every effort was made to avoid asymmetries between the two participants and to make each session as similar as possible. Each participant was assigned a host from the research team to ensure that everything went smoothly from the beginning and that the actual conversation only began with the recording of body movements. Question 1 on the printed cards (see the Materials section) was changed to “How did you become friends?” in the friends condition, as the participants had already met each other.

### Preprocessing

First, the mocap data for each couple was segmented in each of the six questions used to guide the participants talk about the short story. For the data analysis two questions were selected for Study 1, namely the fifth question (in which the manipulation occurs) and the previous question, used as control. For Study 2, we analyzed the fifth question (even though no manipulation occurred), to be able to compare the same question from Study 1.

We exported data from the Motive software supplied by the mocap system manufacturer. Then, we used custom scripts to trajectorize the markers (i.e., to identify the same marker in different frames/at different times and assign it to a single trajectory). Next, we manually labeled corresponding body parts and identified the participant to which each marker belonged. Finally, we visually inspected the results.

As previously mentioned, the manual labeling of markers was necessary because we decided to attach as few markers as possible on the participants' bodies. The resulting 15-markers-per-participant scheme did not directly lend itself to automatic labeling. Sometimes trajectorization failed to follow a marker, resulting in two or more concomitant trajectories that were not recognized as a single trajectory. This trajectorization failure could have occurred during rapid movements or because of artifacts inherent to optical motion capture with several cameras. Because of the nature of this technology, any given marker is not always visible by all cameras at the same time, though, ideally, it is visible in at least six of them. When a marker moves along a smooth trajectory, the set of cameras that capture it may shift. Any set of cameras will detect it in the exact same location if the camera system is calibrated perfectly. In practice, calibration unavoidably presents small measurement errors, and changing camera sets will, on rare occasions, result in an artificial jump of a few millimeters from one measurement of the marker to the next.

As a consequence, two problems arise. First, an actual trajectory ends up as two or more consecutive trajectories. This problem adds to manual labeling efforts because the actual trajectory needs to be labeled several times. However, this manual labeling solves the problem, as equally labeled trajectories can be automatically joined. Second, markers occasionally make short jumps, which translate into a very high instant speed. We solved that problem by applying a single-pole, low-pass digital filter with a 10-Hz cutoff frequency to speed signals. In our observations, this solution removed artifacts without affecting actual motion signals. In addition, we did not expect humans to move faster than that speed in our setting.

A final visual inspection was conducted to reveal any mistakes in manual labeling and trajectory gaps that corresponded to short periods of time in which a marker was not visible to enough cameras to determine its location. Those split-second gaps were closed through linear interpolation. A precondition for interpolation was that the gap was no longer than 0.1.

In each session of Study 2, motion data was automatically segmented by speech turns, allowing the data to be labeled as follows: “Subject A” was always the one speaking, and “subject B” was the one listening. We labeled the data by quantifying motion, as we observed that the speaker could be clearly identified as the one who was moving the most. While in Study 1, the confederate was labeled “Subject A”, and the participant was labeled “Subject B.”

### Computation of speed cross-correlation curves

After preprocessing, our data consisted of a collection of position time series for each recording session. We computed discrete speed signals (distance over time within each measurement period) by taking a marker's position at each frame and subtracting its position at the immediately previous frame^5^. Then we applied a low-pass filter as detailed in the previous section.

Speed is a good choice for this analysis because it tends to be unbiased: unlike position, which tends to remain at a non-zero level (at a distance from an arbitrary origin point), speed tends to have a zero mean, from and to which motion events often depart and return. Such signals are appropriate for correlation analysis. Using position signals and removing the mean is not a good choice in this case because the average position can change over time as participants change poses. Attempting to solve this problem with high-pass filtering would introduce the need for unnecessary design choices (e.g., a cut-off frequency).

Usually, the Pearson correlation between two chosen motion signals from two participants will yield a single number, which will indicate how similar motion signals are, though only for motion events that occur at the same time. We expected some bodily coordination between the participants to occur not only almost immediately but also after a delay. Therefore, we computed cross-correlations, which corresponded to Pearson correlations for every possible delay within a range, including zero delay (i.e., immediacy). The result was not a single number but several organized in the form of a curve.

To describe the analysis, we will name the two participants “A” and “B.” Who is who depends on the study, as detailed in the section above on participants. In our curves, positive lag times reveal body coordinations in which a motion pattern occurs first in participant A and then in participant B. Conversely, negative lags relate to motion patterns that occur first in participant B and then in participant A. In this way, we have information about B's imitations of A, A's imitations of B, and immediate coordination, which has zero delay.

For each participant, we averaged the position of the four back markers and computed a 3D speed signal to represent motion of the torso. Analyzing how the torso motion of two interacting participants relates mostly provides information about proximity, although motion in other body parts also reflects highly attenuated back motion; specially head and arm movement. Although we recognize that interpersonal coordination is a whole-body phenomenon, we are interested in torso motion because it is a good representation of bodily motion which is relevant in its own right, it simplifies our exploration of the already complex phenomenon of human coordination, and we can cleanly measure it with low error since mocap allows us to record it isolated from arms, legs, and head motion. We also expect this technique to allow comparable future research of head and extremities coordination.

Although cross-correlating a pair of 3D signals using vector dot products was possible, we realized that this approach combined different possible motion relationships between participants, making them indistinguishable. We opted for computing regular 1D cross-correlations. We computed these 1D cross-correlations for the axis that goes through both participants the proximity axis, which reflects how participants coordinate while moving closer or farther away from one another.

To compute the speed correlation for a pair of 1D speed signals, (*a, b*), we used the following formula.

(1) $$\begin{array}{r} r_{ab}=\frac{\sum_{i\;=\;1}^{n}\left(a_{i}-\bar{a}\right)\left(b_{i}-\bar{b}\right)}{\sqrt{\sum_{i\;=\;1}^{n}{\left(a_{i}-\bar{a}\right)}^{2}}\sqrt{\sum_{i\;=\;1}^{n}{\left(b_{i}-\bar{b}\right)}^{2}}} \end{array}$$

where $\bar{a}$ and $\bar{b}$ are the average values of *a* and *b*. Since we subtracted average signals before computing correlations, this equation is simplified as follows:

(2) $$\begin{array}{r} r_{ab}=\frac{\sum_{i\;=\;1}^{n}a_{i}b_{i}}{\sqrt{\sum_{i\;=\;1}^{n}a_{i}^{2}}\sqrt{\sum_{i\;=\;1}^{n}b_{i}^{2}}} \end{array}$$

The resulting *r*_*ab*_ in equation (2) corresponds to an immediate correlation (i.e., coordinated motion that occurs at the same time in both participants). To obtain a correlation value for coordination between participants that occurs with a delay Δ*t*, signal *b*, corresponding to the second participant, must be shifted in time by Δ*t*. Notably, as a result, the two signals no longer begin and end at the same time. One end of *a* and the opposite end of *b* need to be trimmed before computing correlations. We produce a cross-correlation curve by computing correlations at several delays, ranging from −1.5 to 1.5 s. We go over that time range in 0.1 s steps, producing a curve with 31 points, which correspond to different time delays (15 negative, 15 positive, and zero). For each analyzed segment for each dyad, a single curve is generated by averaging the four curves obtained from the four back markers. We computed all cross-correlations in time domain and used trimming instead of zero-padding for correlating delayed signals. Therefore, non-periodicity or edge effects are not an issue, nor there is a need for scaling by a windowing function.

#### Aggregation of cross-correlation curves

After applying the previously described process to our data, we were left with one cross-correlation curve for each analyzed segment for each dyad. We needed a statistically sound procedure to aggregate the cross-correlation curves of all participating dyads into a grand average. We approached this problem by rewriting correlation equation (2) as follows:

$$\begin{array}{l} r_{ab}=\frac{s_{ab}}{\sqrt{ss_{a}ss_{b}}} \end{array}$$

Where

$$\begin{array}{l} s_{ab}=\overset{n}{\underset{i\;=\;1}{\sum }}a_{i}b_{i}\\ ss_{a}=\overset{n}{\underset{i\;=\;1}{\sum }}a_{i}^{2}\\ ss_{b}=\overset{n}{\underset{i\;=\;1}{\sum }}b_{i}^{2} \end{array}$$

In short, *ss*_*a*_ is the sum of squares for signal *a*; *ss*_*b*_ is the same for signal *b*; and *s*_*ab*_ is the sum of products. This equation allows us to compute a pooled grand correlation *R*_*ab*_ from *m* correlation values.

$$\begin{array}{l} R_{ab}=\frac{s_{ab,1}+s_{ab,2}+\ldots +s_{ab,m}}{\sqrt{\left(ss_{a,1}+ss_{a,2}+\ldots +s_{ab,m}\right)\left(ss_{b,1}+ss_{b,2}+\ldots +ss_{b,m}\right)}} \end{array}$$

Repeating this calculation for every relevant delay produces a single aggregated cross-correlation curve.

### Statistical inference

Two statistical inference questions were relevant. First, we wanted to know which parts of the curve deviated from zero more than we would expect by chance, given the null hypothesis that no systematic coordination existed between participants. Second, we wanted to know which times displayed statistically significant differences between curves, given the null hypothesis that no differences between two experimental conditions existed. Both questions could be answered by computing and plotting confidence intervals.

We used the Fisher transform *F*(*r*) of correlation *r*, which is suitable for datasets with many degrees of freedom (like ours).

(3) $$\begin{array}{r} F\left(r\right)=\frac{1}{2}\ln\;\frac{1+r}{1-r}=\tanh^{-1}\left(r\right) \end{array}$$

This value is normally distributed with a standard error of $\sigma \approx 1/\sqrt{n}$, where *n* is the sample size. For an aggregated cross-correlation value,

(4) $$\begin{array}{r} \sigma \approx \frac{1}{\sqrt{\sum_{i\;=\;1}^{m}n_{m}}} \end{array}$$

where *n*_*m*_ is the length of the *m*-th signal in the aggregation.

To compute a confidence interval for a correlation value *r*_*ab*_, we first computed an interval for *F*(*r*), which is easier because it is normally distributed with a known standard error. Then, the limits of the interval were converted back to correlation values using the inverse transformation *r* = *F*^−1^(*r*) = tanh(*r*). We chose to set a very conservative α = 0.001. In addition, for our actual signification threshold, we divided that value by 31 to correct for the family-wise error rate in our conclusions, as each curve has 31 points. Due to the many degrees of freedom in our data, confidence intervals were very narrow, even with our conservative approach. The high statistical power of this computation of confidence intervals results from the fact that we do not perform statistical inference on per-subject averages of correlation, which would lose detail.

Based on Pearson correlations, our cross-correlation curves had top magnitudes of approximately 0.15, and they were often approximately 0.05. These values may sound weak for Pearson correlations. However, these values were expected—and could even be considered high—given that more phenomena were occurring in the motion of the two participants than simply coordinated motion. As previously detailed, our strategy was to compute a grand average from several motion data segments, such that consistent coordinative patterns would emerge and time local particularities would generally disappear. Since usual interpretations in terms of correlation magnitudes did not apply, our analysis included testing for statistically significant differences.

### Interpretation of cross-correlation curves

**Figure 1:**
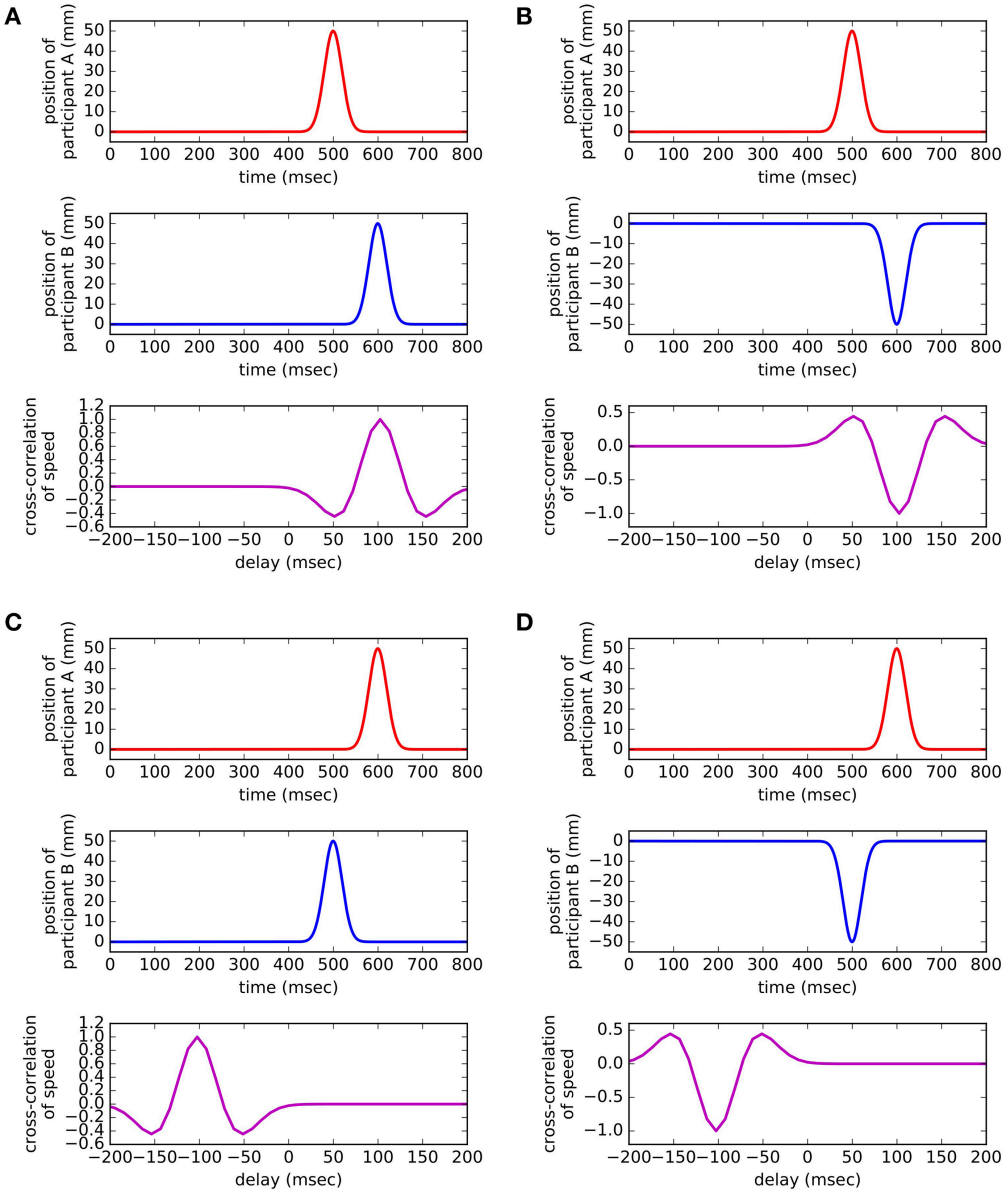
Synthetic examples. Four synthetic data examples show how cross-correlation reflects participants' movements. The four cases shown here can be regarded as motion elements that make up the cross-correlation curve of a full interaction. **(A)** Participant A moves first. B moves 1 s after. This is reflected by cross-correlation peaking at a 1-s delay. **(B)** Although similar to the previous case, B reacts in the opposite direction. Therefore, cross-correlation has a negative peak. **(C)** If B moves before A, the delay has a negative sign. **(D)** Here, B moves first, and A reacts by moving in the opposite direction.

Figure 1 shows artificial motion signals to illustrate how cross-correlation curves reflect different kinds of relationships between the movements of two people. Cross-correlating speed signals yields information about the usual delays between a participant's motion and the other participant's similar reaction and about whether the participants move in the same direction. Notably, cross-correlation provides no information about when participants move in absolute interaction time.

A cross-correlation peak indicates that the two participants performed a similar movement. The delay at which the peak appears in the curve (*x* axis) is the between-participant time difference of those two similar movements. A delay of zero corresponds to the two participants moving at the same time. Positive delays correspond to subject A moving first and then B moving. Negative delays occur when subject B is the first one to move. The magnitude of a peak shows how similar movements were. Negative peaks correspond to similar movements performed in opposite directions. For instance, when A leans forward, B leans back.

Considering the complexity of human motion during interactions, our analysis does not focus on any particular time during the interaction, instead focusing on the process as a whole. The cross-correlation curves show the sum of all the time-localized phenomena described above. Therefore, a peak in the cross-correlation means that participants tend to consistently show bodily coordination in the form of diverse kinds of similar motions. A peak's delay indicates the typical time delay between the two participants for these diverse forms of coordination. The magnitude relates to how frequently this coordination occurred, and the sign indicates whether these movements tended to be in the same or the opposite direction. Thus, positive correlation values correspond to mirror-like coordination between the two participants, while negative correlation values indicate complementary (or anatomical) coordination between the participants.

**Figure 2:**
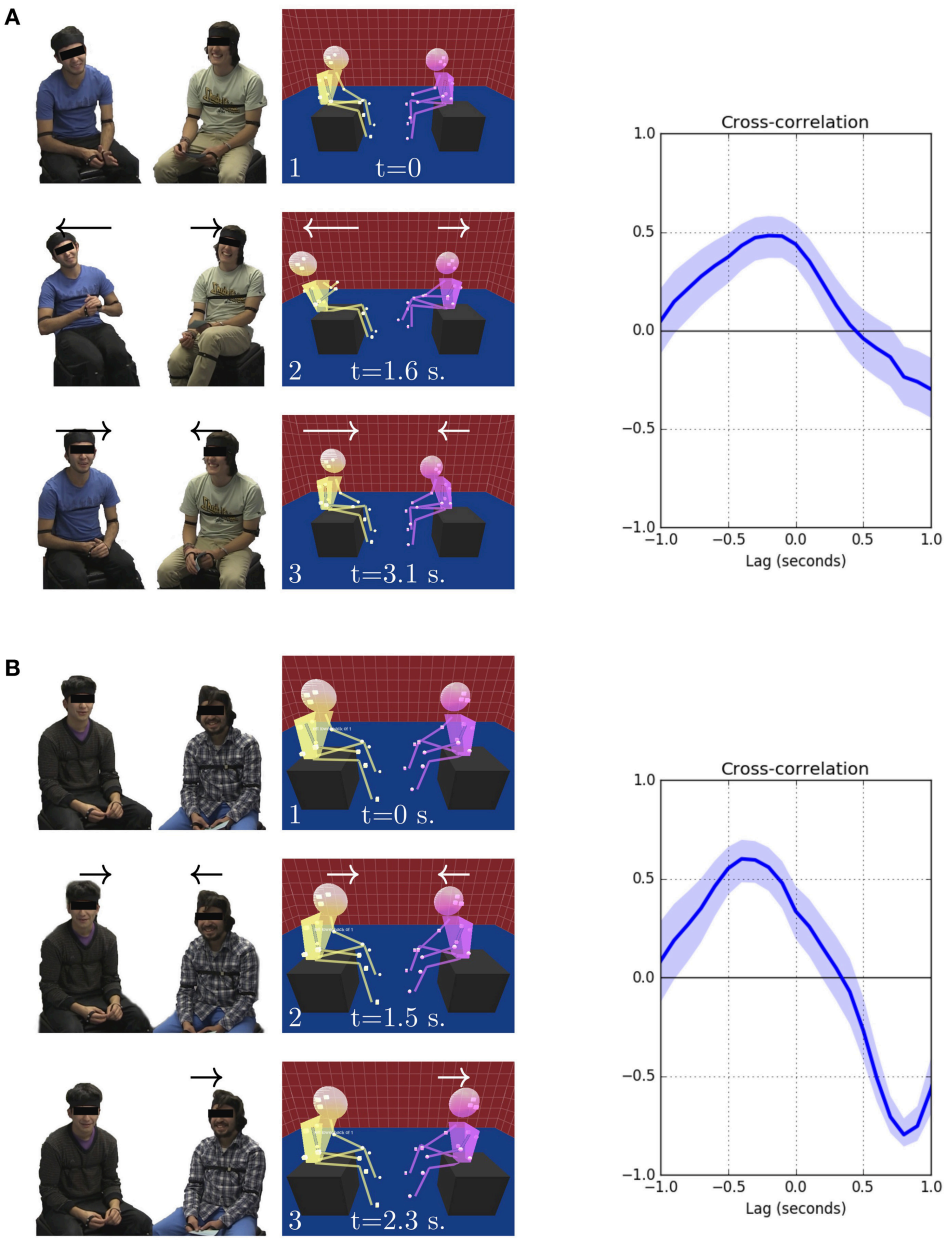
Real data examples. Correlation curves for simple isolated motion events from actual data. Written informed consent was obtained from all participants appearing in this image. Participants are sitting face to face. Note that the timing of the cross-correlation peaks does not relate to absolute time or the beginning of the motion; it instead relates to the lag with which participants move relative to each other. Correlation values are relatively high because each example corresponds to a short piece of recording, which only contains coordinated motion. **(A)** The participant leaned back (frames 1–2) and then returned to their original positions (frames 2–3). The cross-correlation plot displays a positive correlation around zero lag, corresponding to mirrored coordination at almost the same time. Actually, participant B (right) was 0.2 s ahead, which reflects in the curve peaking at a lag of −2 s. **(B)** The participants leaned forward in a subtler motion than that in the previous example (frame 1-2), producing a positive correlation peak at a leg of −0.4 s. Thus, participant B (right) was 0.4 s ahead in time. Thereafter, only participant B (right) continued moving by leaning back (frames 2–3). The leaning forward motion of participant A peaked at 1.5 s (frame 2). The leaning back motion of participant B peaked at 2.3 s (frame 3). Therefore, the curve displays a positive leg (A was 0.8 s ahead) negative correlation (opposite of mirroring) peak at 0.8 s.

To appreciate how this experimental context translates into a real-world situation, Figure 2 shows two isolated motion events between participants, segmented from real capture data. The aggregation of such events produces the final cross-correlation curve for each dyad and ultimately a grand average for an experimental condition. Note the relatively high Pearson correlation values, peaking near *r* = 0.5. These values are high because the correlation curve was computed only for a time segment in which coordination occurred. In a full dataset, these segments share space with other non-coordinated periods, resulting in much lower expected correlation values.

In fact, as the results section will show, we found correlations that would be considered small in a study using questionnaires. However, questionnaires are designed to measure particular constructs as cleanly as possible. By contrast, our motion recordings measured not only the coordinated motion that we wanted to capture but also other kinds of motion occurring in natural interactions. In other words, by a large margin, not all motion is coordinated. Therefore, low correlation values were expected. As detailed in the statistical inference section, we managed to detect consistent coordination from data by computing a grand average from several motion recording sessions and testing for statistically significant differences between conditions and from zero correlation.

Notably, for aggregated cross-correlation curves, the difference in correlation values has two possible causes: the frequent occurrence of a certain coordination pattern or the amplitude of the participants' motion, i.e., how *much* they gesture. In any case, correlation values indicate how *present* a coordination pattern is in an experimental condition.

## Results

### Study 1

Each segment of motion capture data corresponded to a question discussed by two participants (duration: *M* = 435.00 s, *SD* = 315.51 s). Table 1 displays statistics for durations of segments that were analyzed.

**Figure 3:**
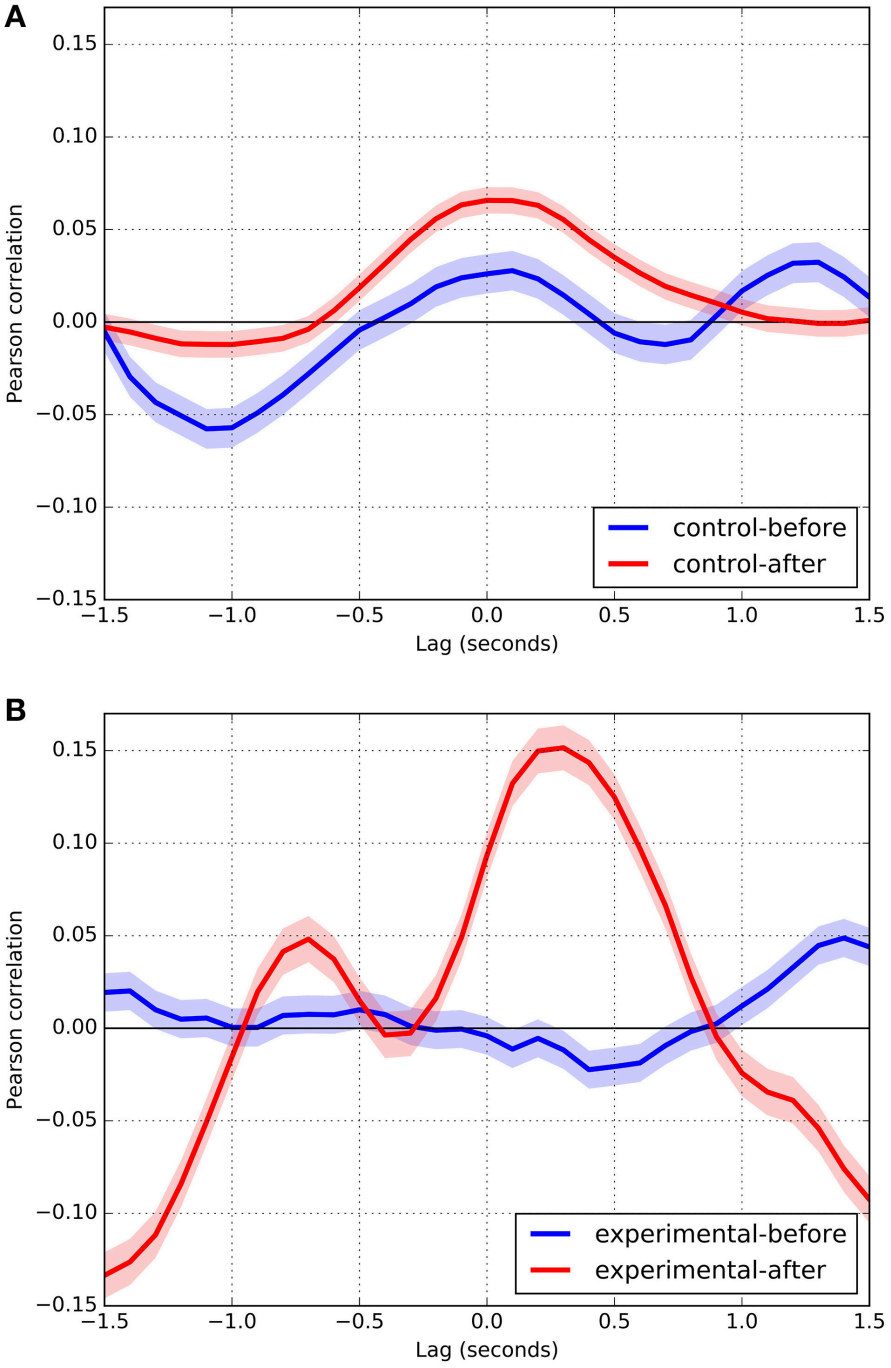
Cross-correlation curves for study 1. The colored area surrounding the curves indicates the confidence interval. The axes have been arranged so that positive correlation values correspond to mirror-like coordination between the two participants. Conversely, negative correlation values indicate complementary (or anatomical) coordination between the participants. Blue and red curves display coordination occurring before and during the breach of trust, respectively. Because there is no breach of trust in the control condition, the blue and red curves correspond to different moments over the course of the interaction (blue earlier, red later). Positive lag times correspond to the participant lagging behind the confederate, i.e., the participant's reactions to the confederate. Conversely, negative lag times correspond to the confederate's reactions to the participant. **(A)** Control Condition. **(B)** Experimental condition.

Figure 3 shows cross-correlation curves for the motion data captured in Study 1. When comparing control to experimental conditions before the trust breach (blue curves in Figures 3A,B), we observe correlations that depart from zero with the same moderate magnitudes but different morphologies. Two correlation peaks are evident in the control condition. The first at *t* = −0.9 s, *r* = 0.033 (*p* < 0.001), indicates that the confederate tends to imitate the participant with a 0.9 s lag. The second, at *t* = 0.3 s, *r* = 0.041 (*p* < 0.001), corresponds to a tendency of the participant to imitate the confederate with a lag that approaches simple reaction time levels, therefore sitting midway between delayed imitation and immediate coordination. A correlation peak occurs in the experimental condition at a similar lag (*t* = 0.2 s) but with a negative magnitude (*r* = −0.044, *p* < 0.001), meaning that the participant reacts to proximity changes of the confederate by opposing them, contrary to mirror-like motion. There is also a tendency for the participant to mirror the confederate with a larger delay (*t* = 1.1 s, *r* = 0.051, *p* < 0.001). As displayed by the confidence interval regions, these four correlation peaks have a statistically significant departure from the zero correlation line (*r* = 0) and show statistically significant differences between control and experimental conditions, except for an imitation performed by the confederate (Figure 3A, blue curve at *t* = −0.9 s). This statement about statistical significance follows from the fact that the α = 0.001 confidence intervals, represented by the thickness of plotted curves, do not overlap or touch the *r* = 0 line at the indicated peaks. Therefore, *p*-value for al such contrasts is <0.001.

The same comparison during the trust breach (red curves in Figures 3A,B) yields remarkable differences between the control and experimental conditions. In the latter, a relatively large correlation peak occurs at *t* = 0.2 s (*r* = 0.195, *p* < 0.001), which indicates that the participant tends to mirror the confederate with a very short delay, almost approaching immediacy. At *t* = 0 s, the correlation is still relatively high (*r* = 0.148, *p* < 0.001). The confederate also imitates the participant but opposing his or her changes in proximity, with correlations of relatively high magnitude (*r* < −0.100, *p* < 0.001) with lags that go from *t* = −0.6 s to *t* = −1.5 s and peak at *t* = −1.2 s (*r* = −0.155, *p* < 0.001). In contrast, the control condition displays a somewhat flatter correlation curve, with a negative correlation peak at *t* = 0.2 s (*r* = 0.051, *p* < 0.001).

In summary, as expected, our results show statistically significant differences between the control and experimental conditions during the experimental manipulation (trust breach), where the actual trust breach displays prompt mirrored coordination. Our finding of low but significant correlation levels is not unlike what was found by other coordination studies (e.g., Boker et al., 2002; Ramseyer and Tschacher, 2011). Contrary to expectations, control and experimental conditions also differed before the experimental manipulation, indicating that bodily coordination is affected not only during the actual trust breach but also during its preparation.

### Study 2

**Figure 4:**
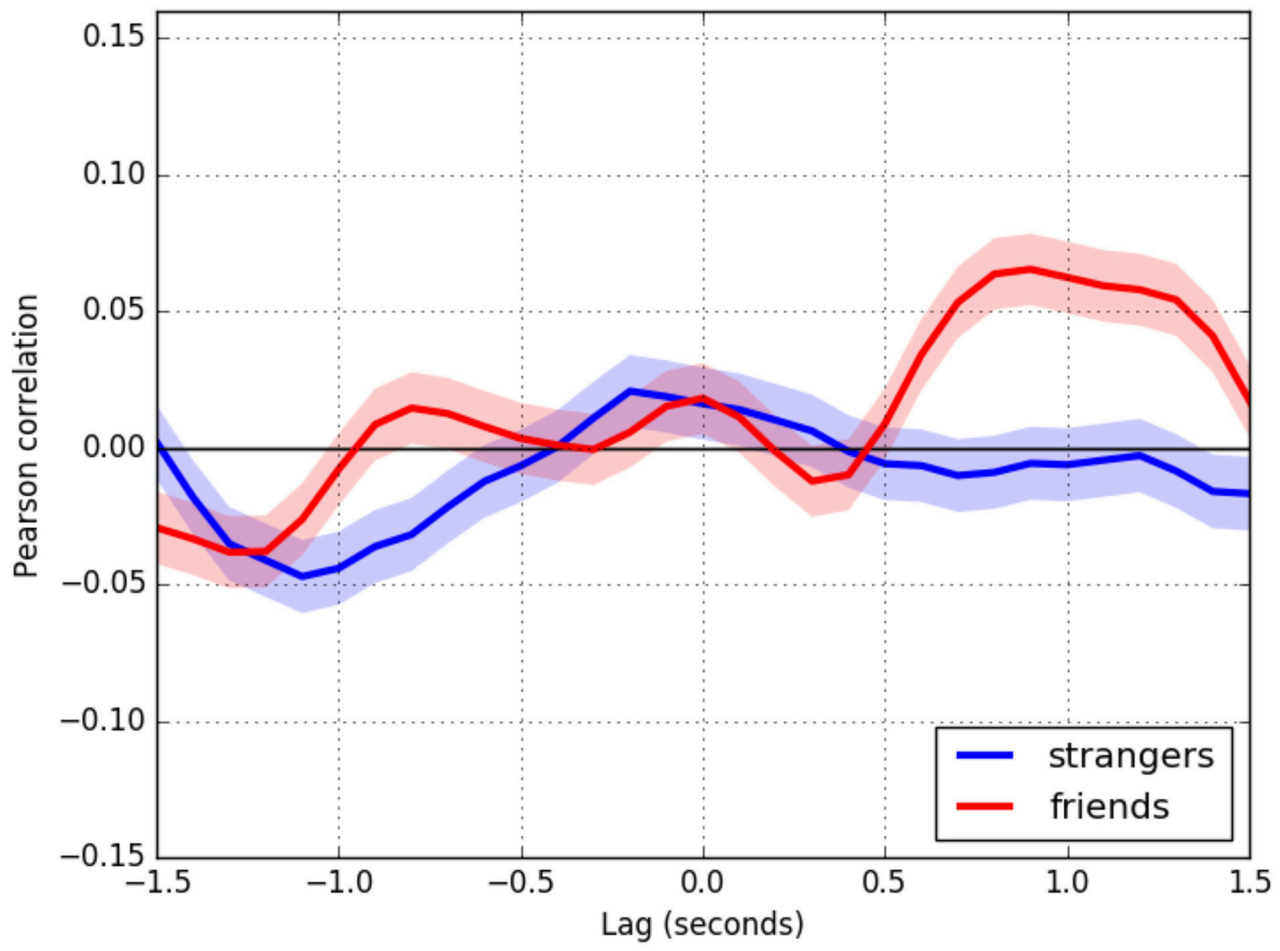
Cross-correlation curves for study 2. As in Study 1, the colored area surrounding the curves indicates the confidence interval; in addition, the axes are arranged so that positive correlation values correspond to mirror-like coordination between the two participants, while negative correlation values indicate anatomic coordination between the participants. Blue curves correspond to stranger dyads, and red curves correspond to friend dyads. Positive lag times correspond to the listener lagging the speaker, thereby reflecting the listener's reactions to the speaker. Conversely, negative lag times correspond to the speaker's reactions to the listener's movements.

Figure 4 displays the cross-correlation curves for study 2. Here, the red curves correspond to interactions between friends, while the blue curves correspond to interactions between people that had never met before. There is no confederate/participant distinction because there are no confederates in Study 2. Data have been organized so that the first motion signal is always from the speaker and thereafter switching to whoever speaks as necessary (see Supplementary Material for a similar analysis of study 1 data). The second motion signal is always from the listener. As a result, the positive lag times in the plots correspond to the listener's reactions to the speaker. Conversely, negative lag times correspond to the speaker's reactions to the listener. The listener mirrors the speaker's movements (*t* = 0.9 s, *r* = 0.065, *p* < 0.001), although only in the friend dyads. A smaller but still statistically significant peak (*t* = −0.8 s, *r* = 0.015, *p* < 0.001) shows that the speaker mirrors the listener with the same time delay in the friend dyads. Finally, there is a negative lag correlation that peaks at (*t* = −1.1 s, *r* = −0.047, *p* < 0.001) for strangers and at (*t* = 1.2 s, *r* = −0.038, *p* < 0.001) for friends.

Generally speaking, correlations found in study 2 were of higher magnitude than those in study 1, but still small. As previously mentioned, this is to be expected and other coordination studies have also found low magnitude correlations (e.g., Boker et al., 2002; Ramseyer and Tschacher, 2011).

## Discussion

The present research aimed to study the effects of trust on attributes that shape interpersonal coordination in natural settings. In pursuit of this goal, an optical motion-capture system was set up to record pairs of participants engaged in natural conversations and to track their spontaneous body movements. Although previous research has focused on the temporal-spatial attributes of interpersonal coordination (Ramseyer and Tschacher, 2011; Paxton and Dale, 2013a,b; Latif et al., 2014; Tschacher et al., 2014), mocap technology additionally makes it possible to explore the morphology of interpersonal coordination. Using this device, we obtained and analyzed information not only about coordination latency (whether it is simultaneous or delayed) and amplitude (whether the coordination is strong or weak), but also about the direction of this coordination (i.e., whether the hearer's movements follow those of the speaker's) and its form (i.e., whether it is mirror-like, when both participants mirror their movements, or whether it is complementary, when both participants move in a coordinated and anatomically coherent way). As a result, we were able to provide additional findings to the naturalistic line of studies on socio-contextual factors in interpersonal coordination (Ramseyer and Tschacher, 2011; Paxton and Dale, 2013a,b; Latif et al., 2014; Tschacher et al., 2014).

We conducted two experiments in which we manipulated trust, through a breach of expectancy (Study 1) and friendship (Study 2). Based on reports indicating that affective factors modulate interpersonal coordination (Miles et al., 2010) and that a violation of expectations usually leads to social tension and distrust (Ma et al., 2015), we expected in both cases to find more coordination between participants in conditions involving trust than in situations of distrust. Nevertheless, in Study 1 (see Figure 3), the positive correlation peak in the experimental condition (non-trust) during the breach of trust (red curve) demonstrates the participant tends to coordinate to a greater degree with the confederate after the breach of trust. These results show that, contrary to our prediction, the violation of the participant expectancy leads to even more coordination with the confederate. Although our results are inconclusive, it seems reasonable to suggest that participants became more emotionally involved in the interaction after the breach of trust, which then enhanced coordination between them.

Considering that coordination occurs rapidly–the peak is about 200 ms after the speaker's movements–what we probably see in these results is an initial non-conscious reaction of imitation by the participant as a response to social exclusion involved in the confederate's disclosure. Derfler-Rozin et al. (2010) showed that people who were told that they were likely to be excluded were more trusting toward others compared to a control condition. They suggest that “people who are at risk of being excluded were trying to ‘fix’ their social situation by trusting others, thereby setting up a possibility for reciprocity” (Derfler-Rozin et al., 2010, p. 146). Additional studies report an increased non-conscious imitative behavior by excluded people to recover their group membership (Lakin et al., 2008; Lakin and Chartrand, 2013):“When people are confronted with social exclusion they often strive to reconnect with others by engaging in various behaviors aimed at repairing their feelings of social inclusion and acceptance” (Cheung et al., 2015, p. 1). In line with this evidence, our results may be interpreted as an attempt to recover her sense of belonging to a common group with the interaction partner. In addition to this effect, elicited by the hearer's imitation of the speaker's movements, Figure 3B shows a less pronounced, but discernible, negative correlation peak denoting the confederate's coordination in response to the participant's movements. The later, and less pronounced, mirror coordination perhaps indicates the confederate's attempt to regain the participant's trust and resume the interaction after he/she performed the breach of trust. It would indicate that despite the instructions issued to them, confederates tend to reconstruct the social bond damaged by his/her previous performance.

Moreover, the described positive correlation peak shows the participant's strong early mirror coordination in response to the confederate. This finding is consistent with interpretations of the distinction between mirroring and anatomical forms of imitation derived from developmental studies on mimicry: while mirroring corresponds to a faster and more automatic form of imitation, anatomical coordination appears slightly more delayed in time and is associated with a cognitive perspective taking by the hearer of the speaker (Wapner and Cirillo, 1968; Bekkering et al., 2000; Avikainen et al., 2003; Erjavec and Horne, 2008; Chiavarino, 2012; Barchiesi and Cattaneo, 2013; Pierpaoli et al., 2014; Ubaldi et al., 2015). Interestingly, our data show that mirror coordination is not always imitative, but can also be simultaneous. While the imitative coordination, observed in the positive time lags in Figure 3, denotes the participant closely following the confederate's movements, simultaneous coordination shows immediate synchrony between the movements of the participant and the confederate.

The unexpected interference with the confederate's coordinative movements reflects the high contextual sensitivity of interpersonal synchrony. Even subtle contextual variables can indeed influence the coordinative patterns between people. To similar conclusions arrive recent research into joint actions. For instance, Abney et al. (2015) argued against the predominant view that more coordination is always beneficial for obtaining a common goal. By studying dyads performing a joint task involving the construction of towers from dough, they found that weaker synchronization between subjects' body movements led to better performance than robust synchronization. Wallot et al. (2016) implemented a joint task (to build model cars using Lego pieces) and found that synchronization during joint action is not always beneficial for performance. Crucially, they maintain that the role of synchronization varies depending on the task context. Even though our study was designed to observe coordination in natural conversations, rather than measuring its influence on task performance, our results also show that interpersonal coordination can change rapidly and significantly, depending on the interactional context. We found that latency, amplitude, direction, and form of coordination vary strongly, depending on contextual cues that can even change during the course of the conversation.

In Study 2, we expected greater coordination in couples of friends than in couples of non-friends, since trust pre-exists among friends whereas it must be built up during interactions among non-friends. In line with our hypothesis, the results revealed that imitative coordination tends to be more pronounced in pairs of friends than in pairs of non-friends. This overall result is consistent with the findings of Latif et al. (2014), who found statistically significant differences in the imitative coordination of couples of real friends and couples of strangers. Their results showed higher imitative coordination between real friends than between strangers, suggesting that “friends and strangers conversations contain coordination content unique to their affiliation categorization” (Latif et al., 2014, p. 3). Moreover, these results are in line with investigations conducted within the experimental contexts of dyadic conversations and psychotherapy settings. These studies reveal that interpersonal coordination patterns are sensitive to the qualities of relationships, such as affect, linking, familiarity and rapport (Grahe and Bernieri, 1999; Nagaoka et al., 2007; Nagaoka and Komori, 2008; Ramseyer and Tschacher, 2011; Paxton and Dale, 2013a; Tschacher et al., 2014). Our findings indicate that interpersonal coordination patterns are also sensitive to another quality of relationships: the trust between interacting people.

Another significant finding of Study 2 concerns the bidirectional dynamics involved in body movements during conversational contexts. In line with previous research (Paxton and Dale, 2013c), we found that listeners coordinate their movements with those of speakers, and vice versa, both simultaneous and delayed. This finding indicates that speakers and listeners are mutually attentive to their interlocutor and continuously coordinate their own movements. According to Paxton and Dale (2013c, p. 336), this simultaneous or delayed feedback between speakers and listeners “means that body movement synchrony has properties that differ from synchrony in speech, (…) due to turn-taking.” This interactive dynamics implies complex processes of coupling and mutual adaptation, showing that the roles of speaker and listener change constantly and subtly over the course of an interaction. Thus, the speaker is not only acting in front of a passive listener, but is also reacting to a somewhat active listener, who is continually moving and providing feedback.

In particular, the simultaneous coordination reported here eloquently shows the ongoing co-adaptation of movements between the two participants in the course of their interaction. Interpersonal coordination in natural human interactions deploys rapid and dynamic patterns of mutual adaptation among interacting parties, whose functions remain to be studied. This finding reinforces the insight from naturalistic studies on interpersonal coordination–to focus not only on unidirectional interactions, in which one participant acts/speaks and the other only reacts/listens, but crucially also on dialogical interactions where people are in a state of constant mutual adaptation (LaFrance, 1977; Bernieri et al., 1988; Boker et al., 2002; Paxton and Dale, 2013a,b; Latif et al., 2014; Tschacher et al., 2014).

Importantly, the analysis from Study 2 not only confirms previous findings, but also presents novel insights into the form of this coordination. We found, additionally, that the imitative coordination between pairs of friends is predominantly mirror-like, whereas between pairs of non-friends it is primarily anatomical; imitative mirror coordination is observed in the delayed positive correlation peaks shown in Figure 4, while anatomical coordination is observed in the delayed negative correlation peaks. The presence of these morphologically different coordinating patterns suggests that trust between individuals modulates their imitative bodily dynamics differentially during a conversation. We consider that the effect of trust on these patterns of coordination of friends and non-friends can be related to psychological dispositions specific to each type of relationship.

Evidence from developmental studies on imitation indicates that there is a rapid and spontaneous tendency in children and adults to imitate in a mirror-like way rather than anatomically (Wapner and Cirillo, 1968; Erjavec and Horne, 2008; Dunphy-Lelii, 2014; Ubaldi et al., 2015). Furthermore, there is evidence of an association between high error rates in mirror-like imitations and emotional competence deficits in individuals diagnosed with autistic spectrum disorders (Avikainen et al., 2003). These results suggest that this imitative modality is involved in affectively guided interactions (Avikainen et al., 2003; Dunphy-Lelii, 2014). Additionally, the associations found between lower error rates in anatomical imitations and higher perspective-taking scores indicate this imitative modality is involved in tasks of an intellectual or cognitive nature (Gleissner et al., 2000; Jiménez et al., 2012; Sudo et al., 2012; Pierpaoli et al., 2014).

Although our results are not conclusive, the aforementioned evidence prompts that the mirror-like imitative coordination predominantly observed in relationships between friends corresponds to a manifestation of an affective disposition toward the other underlying such interactions, since a less self-monitored interaction should manifest in mirror-like forms of coordination. Conversely, the anatomical coordination observed in relationships among non-friends indicates a more intellectual disposition to interact. These interpretations should be explored in future investigations.

According to Tarr et al. (2016), synchronization between people changes in situations in which an individual disconnects from interactions with others to concentrate on satisfying a role. This applies, in our interpretation, to the differences observed between the conversations before the bearch of trust in the control and experimental conditions of Study 1 (blue curves in Figures 3A,B). Considering that both situations represent interactions between individuals who previously did not know each other, where the same question is answered and where no breach of trust has yet occurred, similar curves were expected. Nevertheless, this was not the case. Presumably, the task assigned to the confederate prevented her/him from interacting naturally with the participant, what manifests in a rather plain curve in the experimental condition even before the breach of trust (blue curve Figure 3B). It may be the case that the confederate was dividing attentional resources between the cognitive task made by the research team and the interaction with the participant, even when no manipulation was required. Our results contribute to the understanding of interpersonal coordination dynamics in a trust context, and extend previous findings. Nonetheless, they are not conclusive; therefore, further research on interpersonal coordination in natural and non-natural contexts, as well as investigation of its psychological functions, is required.

### Limitations of the studies

Some limitations of both studies should be mentioned. First, it has to be noted that we assumed in Study 2 that friendship is associated with trust. Previous studies also adopt this assumption (e.g., Latif et al., 2014), which is based on evidence showing that people have greater trust in their friends than in strangers (Glaeser et al., 2000; Marzoli et al., 2011; Binzel and Fehr, 2013; Launay et al., 2013). However, it is also true that both conditions (friends and non-friends) differ in other aspects besides trust (e.g., the similarity between interactants, empathy, reciprocity feelings, among others). Future research might distinguish those additional factors of friendship from trust effects.

Furthermore, in Study 2, we found that trust influences interpersonal coordination. However, previous studies have demonstrated the inverse relationship, i.e., that synchronization increases trust (Launay et al., 2013). Since Study 2 does not provide information on the causal relationship between friendship and coordination, we did not explore the question if friendship causes more imitative coordination or rather the reverse. Future research should address the reciprocal relationship between trust, interpersonal coordination, and friendship.

In general terms, it is relevant to underline that, even though a mocap system is currently the most accurate technique for tracking human movement, it cannot wholly replicate natural interaction. A mocap system ultimately requires markers to be positioned on subjects' bodies and tracked, and conversations to be elicited in an experimental setting. Such restrictions are less marked when using video techniques. Of course, mocap data facilitate different kinds of analysis that cannot be performed using video analysis. Further research should focus on whether this gain in accuracy and analytical power compensates for the reduced naturality of data obtained in this way (Romero et al., 2017).

A related limitation of mocap studies is precisely the need to reduce marker information on account of the massive amount of data obtained from a design aimed to capture natural conversations. We offer here a subset of data, focused on the torso movements of interactants, leaving information relating to other limbs unanalyzed. Future work should take into account this information, which requires different analysis procedures because of the different types of movement associated with the arms, hands and head. Finally, our results highlight the pervasive effect of subtle instructions to the interactants on their movement coordination. This also applies to the role of the confederates, whose motion patterns were involuntarily affected by the task assigned to them. One way to reduce this interference could be to instruct the confederate only at the moment of the breach of trust–for example, by employing a hidden ear piece.

## Conclusions

We presented evidence of the impact of trust on interpersonal coordination. Breaching trust during a natural conversation produced a radical modification of coordination patterns between interactants; the participants exhibited immediate high mirror-like coordination, as did the confederates, although in a more moderate way. We also showed that, in line with previous research, interpersonal coordination is more prominent between friends than between strangers. The fact that the form of this coordination was mirror-like suggested an affective, rather than an intellectual, involvement.

Our studies confirm that it is possible to use new technologies such as mocap to enhance our knowledge of real social interactions. To process the mocap data, we conducted analyses suited to the nature of the phenomenon: interactional and continuous along time. Consequently, our analysis provided information about the intensity and delay of coordination. More importantly, it provided information on who imitates whom, and what form coordination adopts. Thus, we were able to identify two forms of coordination, mirror-like and anatomical, which have been described in the literature as satisfying different communicative functions.

To conclude, our results indicate the high contextual sensitivity of interpersonal coordination. The use of more accurate techniques sheds light on the complexity of interpersonal coordination throughout the course of a real conversation. Rather than being an on/off feature of human interaction, our studies reveal that interpersonal coordination adopts different intensities, temporal coincidence, and morphology. It varies rapidly and continuously during a conversation, depending on contextual cues such as the dispositional attitude toward the other. This finding, in sum, implies that future research should consider not only the presence/absence of interpersonal coordination, but also its form and time evolution.

## Data availability statement

The full datasets used for this manuscript are not publicly available because of restrictions imposed by the informed consent assigned by participants. Requests to access the datasets should be directed to Carlos Cornejo, cca@uc.cl.

## Ethics statement

All subjects gave written informed consent in accordance with the Declaration of Helsinki. The protocol was approved by the Ethical Committee of Social Sciences at the Pontificia Universidad Católica de Chile.

## Author contributions

All authors of this article satisfy the authorship criteria. CC, EH, DC, HO, and JR made contributions to the conception of the paper, data collection, data analysis, and participated in the writing process by adding substantively relevant content. CC, EH, ZC, AT-A, and JR participated in data collection, data analysis as well in the writing process. All authors approved the final version to be published. They all agree to be accountable for all aspects of the text in ensuring that questions related to the accuracy or integrity of any part of the work are appropriately investigated and resolved.

### Conflict of interest statement

The authors declare that the research was conducted in the absence of any commercial or financial relationships that could be construed as a potential conflict of interest.

## Footnotes

^1^Available online at: http://optitrack.com/products/prime-41/

^2^Available online at: http://optitrack.com/products/motive/

^3^It would be perfectly possible to replicate this study by using any motion capture technique that allows measurement in a single dimension, as long as (1) it is of similar or better precision, (2) the single axis of measurement is aligned with the proximity axis of the participants, and (3) it is capable of isolating torso motion from head and extremities. Our analysis focuses on torso motion alone, as indicated in the Preprocessing section.

^4^This short fictive story is a description of the afterlife of anyone, in which the total time spent on ordinary activities (such as sleeping, waiting, and driving) is summed up. The tale is profound, though slightly humorous. This story was chosen because it elicits personal reflections with no strong emotional commitment. Furthermore, as a fiction, it does not produce asymmetry in information between participants.

^5^In the context of our calculation of cross-correlation curves, it is unnecessary to divide position differences (distances) by measurement period length (time), since this only has the effect of linearly scaling all speed values by the same factor (1/framerate). Such scaling makes no difference after computing Pearson correlations. This is still speed, since it is the distance a marker travels in the time between each frame and the next.
