# Supplementary material for: Dynamics of Simultaneous and Imitative Bodily Coordination in Trust and Distrust
Source: Front Psychol. 2018 Aug 28;9:1546. doi: 10.3389/fpsyg.2018.01546 (PMC6121516; doi:10.3389/fpsyg.2018.01546)
Supplement: Supplementary file 1 [file Data_Sheet_1.PDF]

Supplementary Material

Dynamics of Simultaneous and Imitative Bodily Coordination in Trust and Distrust

Carlos Cornejo [1,*], Esteban Hurtado [1], Zamara Cuadros [1], Alejandra Torres [1], Javiera Paredes [1], Himmbler Olivares [2], David Carré [3], Juan Pablo Robledo [4]

[1]*Laboratorio de Lenguaje Interacción y Fenomenología, Escuela de Psicología, Pontificia Universidad Católica de Chile, Santiago, Chile*

[2]*Departamento de Psicología, Universidad de Concepción, Concepción, Chile*

[3]*Carrera de Psicología, Universidad Nacional Arturo Prat, Iquique, Chile*

[4]*Centre for Music and Science, University of Cambridge, Cambridge, UK*

***Correspondence**: Carlos Cornejo: cca@uc.cl

1. Additional data and plots

By design, our two studies followed slightly different approaches to the generation of bodily motion cross-correlation curves. In study 1, each recording session was a conversation between a naive participant and a confederate. Consequently, each motion cross-correlation signal was computed between a participant and a confederate. Study 2 only included naive participants, which allowed us to study a more symmetric relationship, but left us with no participant/confederate distinction. Consequently, as detailed in our methods section, we classified data segments so that we could compute each cross-correlation between a speaker and a listener.

This speaker/listener distinction was not considered as an additional analysis approach for study 1 data, since the participant/confederate distinction covered the interest we had wen we designed that study. Also, since the relationship is not symmetric (there is a confederate), a speaker/listener cross-correlation curve plot would be hard to interpret, because we know there is a very important underlying participant/confederate distinction and it has no representation in such a plot. It was kindly brought to our attention that, regardless of this issue, readers of our article could be interested in additional cross-correlation curves for study 1, analyzed by the same speaker/listener approach used in study 2. We include that plot as supplementary material.

For completeness, here we also include full numeric data that was represented in our plots.

2. Speaker/listener cross-correlation plot for study 1

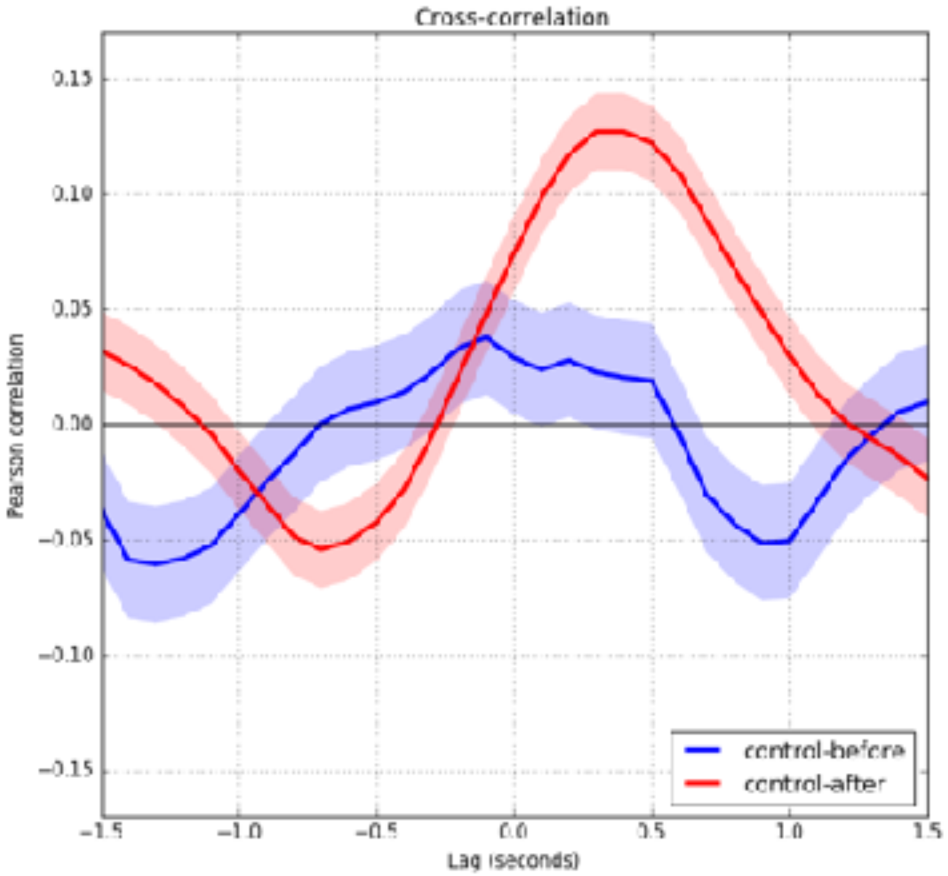

Supplementary figure 1. Cross-correlation curve for control condition motion signals recorded in study 1, but computed according to the exact same procedure of study 2. Even though statistically significant different patterns occur between both experimental conditions, it is important to remember that each curve in this particular plot includes data from both confederates and naive participants. Therefore, this curve does not represent confederate/participants dynamics, but is implicitly affected by them. This makes it difficult to interpret this curves in a way that addresses our original research questions. This difficulty is absent in the two plots we included in the article, because such distinction does not exist in study 2, and it is explicitly addressed in our plot for study 1.

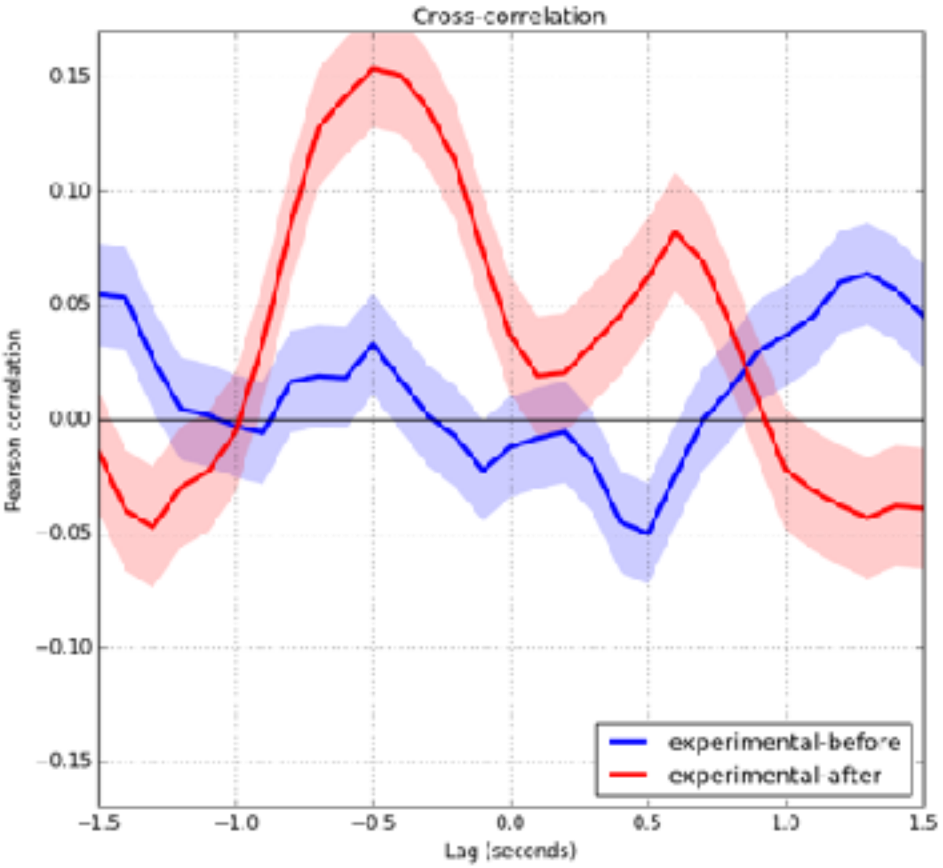

Supplementary figure 2. Cross-correlation curve for experimental condition motion signals recorded in study 1, but computed according to the exact same procedure of study 2. This plot complements supplementary figure 1.

3. Supplementary Data

In the following tables, each row corresponds to a point in a plot. Time and standard errors are in units of seconds. Column variables are as follows.

- **Curve**. Name of the curve as shown in the plot's legend.
- **Time**. Time (lag) in seconds for the correlation value point.
- **Correlation**. Pearson correlation value.
- **Fisher Z**. Correlation after Fisher Z transformation for construction of confidence intervals. Note similarity with *z*-values. This is a consequence of the fact that arctanh(*x*) function can be approximated by *x* when *x* is small.

- **Degrees of freedom**. Roughly corresponds to the amount of recorded values included in the computation of the correlation value.
- **p-value**. Statistical significance of a Fisher Z test on correlation. Null hypothesis is that correlation is zero.

3.1. Study 1 cross-correlation plot data (Figure 3)

3.1.1. Control condition (Figure 3a)

CURVE	TIME	CORRELATION	DOF	FISHER_Z	P-VALUE
**control-before**	-1.5	-0.00526348319644	-0.00526353180421	49230	0.242861738056
**control-before**	-1.4	-0.0296115870443	-0.0296202465353	49338	4.7268028411E-11
**control-before**	-1.3	-0.043340665019	-0.0433678328714	49446	5.23868899776E-22
**control-before**	-1.2	-0.0505637570455	-0.0506069152804	49554	1.94317591783E-29
**control-before**	-1.1	-0.057713930586	-0.0577781386918	49662	6.15637439691E-38
**control-before**	-1	-0.0571040477972	-0.0571662391903	49770	2.98725082887E-37
**control-before**	-0.9	-0.0490838969401	-0.0491233721337	49878	5.27383968921E-28
**control-before**	-0.8	-0.0393311655533	-0.0393514653902	49986	1.39247279222E-18
**control-before**	-0.7	-0.0279210759264	-0.0279283349532	50094	4.08269099096E-10
**control-before**	-0.6	-0.0161401680087	-0.0161415697594	50202	0.000298442372216
**control-before**	-0.5	-0.00431052011337	-0.004310546811	50310	0.333618554303
**control-before**	-0.4	0.00255911560878	0.00255912119541	50418	0.565545706709
**control-before**	-0.3	0.00982075212908	0.00982106787527	50526	0.0272736636992
**control-before**	-0.2	0.0190051301557	0.0190074188375	50634	1.89386813259E-05
**control-before**	-0.1	0.0239051871221	0.0239097422873	50742	7.20810457229E-08
**control-before**	0	0.0260691873813	0.0260750953524	50850	4.1046593697E-09
**control-before**	0.1	0.0277791125998	0.0277862614302	50742	3.87133345698E-10
**control-before**	0.2	0.0233530157974	0.0233572624801	50634	1.47341401246E-07
**control-before**	0.3	0.0145483676098	0.0145493941517	50526	0.00107391233897
**control-before**	0.4	0.00437786562961	0.00437789359823	50418	0.325602435431
**control-before**	0.5	-0.00596007033007	-0.00596014090365	50310	0.181270594181
**control-before**	0.6	-0.0106445614625	-0.0106449635231	50202	0.0170749649757
**control-before**	0.7	-0.0121263142742	-0.012126908708	50094	0.00664346425212
**control-before**	0.8	-0.00957968007272	-0.00957997313214	49986	0.0322057803914
**control-before**	0.9	0.00340114097933	0.00340115409395	49878	0.447498040309

**control-before**	1	0.0168084325015	0.0168100156951	49770	0.000176708738004
**control-before**	1.1	0.0252600217211	0.0252653963219	49662	1.79806102283E-08
**control-before**	1.2	0.0317845588599	0.0317952688895	49554	1.46399361355E-12
**control-before**	1.3	0.0323083349906	0.03231958349	49446	6.63613888096E-13
**control-before**	1.4	0.0243510027138	0.0243558175758	49338	6.30413322416E-08
**control-before**	1.5	0.0134632817123	0.0134640952521	49230	0.00281369451038
**control-after**	-1.5	-0.0027986042599	-0.00279861156634	111087	0.350939095238
**control-after**	-1.4	-0.00535442604404	-0.00535447721517	111219	0.0741491315082
**control-after**	-1.3	-0.00875769358732	-0.00875791749447	111351	0.00347283217249
**control-after**	-1.2	-0.011775190789	-0.0117757350644	111483	8.43092388683E-05
**control-after**	-1.1	-0.0120580670963	-0.0120586515495	111615	5.60969803397E-05
**control-after**	-1	-0.0121245073231	-0.0121251014913	111747	5.05111200185E-05
**control-after**	-0.9	-0.0105827737963	-0.0105831688957	111879	0.000400290770846
**control-after**	-0.8	-0.0087672484831	-0.00876747312394	112011	0.00334298978229
**control-after**	-0.7	-0.00384003283485	-0.00384005170987	112143	0.198461468297
**control-after**	-0.6	0.0062034504229	0.00620353000011	112275	0.0376499504015
**control-after**	-0.5	0.0185979838226	0.0186001285222	112407	4.48632784849E-10
**control-after**	-0.4	0.0315157660707	0.0315262065701	112539	3.84867560639E-26
**control-after**	-0.3	0.0445503677925	0.0445798765028	112671	1.26387494811E-50
**control-after**	-0.2	0.0558170253263	0.0558751006575	112803	1.42470516204E-78
**control-after**	-0.1	0.0633042753907	0.0633890418128	112935	1.07859269282E-100
**control-after**	0	0.0657487972621	0.0658437856799	113067	1.29486037747E-108
**control-after**	0.1	0.0656396394879	0.0657341547682	112935	3.90135969091E-108
**control-after**	0.2	0.0630795138937	0.0631633791954	112803	7.06894899182E-100
**control-after**	0.3	0.0553629927138	0.055419660614	112671	3.07004693195E-77
**control-after**	0.4	0.0444933980173	0.0445227935779	112539	1.92206430618E-50
**control-after**	0.5	0.0349472111846	0.0349614487169	112407	9.88134835647E-32
**control-after**	0.6	0.0264810551808	0.0264872477003	112275	6.98050831644E-19
**control-after**	0.7	0.0192937312866	0.0192961258395	112143	1.03437362357E-10
**control-after**	0.8	0.0145128464547	0.0145138654952	112011	1.18872962173E-06
**control-after**	0.9	0.0102084496404	0.0102088042784	111879	0.000638585429258
**control-after**	1	0.00536302904266	0.00536308046084	111747	0.0730046417874
**control-after**	1.1	0.00184680313416	0.00184680523378	111615	0.53723733602

**control-after**	1.2	0.000590498529839	0.000590498598472	111483	0.843700773706
**control-after**	1.3	-0.000698669566495	-0.000698669680177	111351	0.815651792406
**control-after**	1.4	-0.000717886806241	-0.000717886929565	111219	0.810786286352
**control-after**	1.5	0.000909882152045	0.000909882403138	111087	0.761690793422

3.1.2. Experimental condition (Figure 3b)

CURVE	TIME	CORRELATION	DOF	FISHER_Z	P-VALUE
**experimental-before**	-1.5	0.0193501214903	0.0193525371036	53834	7.11544352993E-06
**experimental-before**	-1.4	0.0201406624493	0.0201433864406	53930	2.89877121817E-06
**experimental-before**	-1.3	0.00999971913454	0.0100000524598	54026	0.020106123724
**experimental-before**	-1.2	0.00488937437759	0.00488941333991	54122	0.25533778773
**experimental-before**	-1.1	0.00552176455477	0.00552182067512	54218	0.198532896526
**experimental-before**	-1	0.000450446089801	0.000450446120266	54314	0.916393179367
**experimental-before**	-0.9	0.000421482132422	0.00042148215738	54410	0.921682388031
**experimental-before**	-0.8	0.00690524505155	0.00690535480759	54506	0.106927116832
**experimental-before**	-0.7	0.00750766716138	0.00750780822287	54602	0.0793697198259
**experimental-before**	-0.6	0.00727958467071	0.00727971326224	54698	0.0886522244273
**experimental-before**	-0.5	0.00997385564002	0.00997418638548	54794	0.0195554735095
**experimental-before**	-0.4	0.00740787449565	0.00740801000645	54890	0.0826356224374
**experimental-before**	-0.3	0.00109823797251	0.00109823841405	54986	0.796772009076
**experimental-before**	-0.2	-0.00107503568268	-0.00107503609682	55082	0.800804407593
**experimental-before**	-0.1	-0.000416199382321	-0.000416199406353	55178	0.922118716174
**experimental-before**	0	-0.0041096785365	-0.00410970167348	55274	0.333939982228
**experimental-before**	0.1	-0.011280022839	-0.0112805012948	55178	0.00805419320441
**experimental-before**	0.2	-0.00545022963182	-0.00545028359914	55082	0.200841671448
**experimental-before**	0.3	-0.0116912048345	-0.0116917375461	54986	0.00611395362584
**experimental-before**	0.4	-0.0223820900318	-0.0223858286509	54890	1.56545646053E-07
**experimental-before**	0.5	-0.0207120300851	-0.0207149925865	54794	1.24091700987E-06
**experimental-before**	0.6	-0.0187458778824	-0.0187480741623	54698	1.16132255311E-05
**experimental-before**	0.7	-0.00934214528817	-0.00934241708309	54602	0.0290321856318
**experimental-before**	0.8	-0.00176526478068	-0.0017652666143	54506	0.68024536882
**experimental-before**	0.9	0.0024038798859	0.0024038845163	54410	0.574982342538
**experimental-before**	1	0.0120151960331	0.0120157742742	54314	0.00510519775607

**experimental-before**	1.1	0.0211329996904	0.0211361465588	54218	8.58830597988E-07
**experimental-before**	1.2	0.0328334169544	0.0328452230971	54122	2.15278021968E-14
**experimental-before**	1.3	0.0446899648415	0.0447197520385	54026	2.62846229287E-25
**experimental-before**	1.4	0.0487680479132	0.048806765226	53930	8.87246877148E-30
**experimental-before**	1.5	0.0439401017267	0.0439684133927	53834	1.94993598721E-24
**experimental-after**	-1.5	-0.133502832282	-0.134304564073	36084	1.44299145478E-143
**experimental-after**	-1.4	-0.126259772133	-0.126937188114	36192	7.69058235215E-129
**experimental-after**	-1.3	-0.111640193861	-0.11210750388	36300	3.19234568824E-101
**experimental-after**	-1.2	-0.0841198835886	-0.0843191453861	36408	3.05530770342E-58
**experimental-after**	-1.1	-0.0505869930023	-0.0506302108242	36516	3.85061347517E-22
**experimental-after**	-1	-0.0154045102097	-0.0154057288745	36624	0.00319572675437
**experimental-after**	-0.9	0.0196099996158	0.0196125138847	36732	0.000170692844032
**experimental-after**	-0.8	0.0413812725966	0.0414049174597	36840	1.90842147386E-15
**experimental-after**	-0.7	0.0481593107172	0.0481965948868	36948	1.96391941879E-20
**experimental-after**	-0.6	0.0373103246877	0.0373276519033	37056	6.693778362E-13
**experimental-after**	-0.5	0.0149217976136	0.0149229052576	37164	0.00401688492293
**experimental-after**	-0.4	-0.00367263769283	-0.00367265420547	37272	0.478298994015
**experimental-after**	-0.3	-0.00260801474616	-0.0026080206592	37380	0.614098133973
**experimental-after**	-0.2	0.0162148779281	0.0162162992365	37488	0.00169080317328
**experimental-after**	-0.1	0.0490068892368	0.0490461787455	37596	1.90844025245E-21
**experimental-after**	0	0.0935135962028	0.0937876210911	37704	4.20410014649E-74
**experimental-after**	0.1	0.13230802793	0.133088275218	37596	7.71193535973E-147
**experimental-after**	0.2	0.149793613886	0.15092930593	37488	9.99648539338E-188
**experimental-after**	0.3	0.151541774012	0.152718072914	37380	1.3207750904E-191
**experimental-after**	0.4	0.143520786979	0.144518571944	37272	2.61681307175E-171
**experimental-after**	0.5	0.124862548569	0.125517583377	37164	2.37877322885E-129
**experimental-after**	0.6	0.0970625805971	0.0973691288272	37056	2.18778086678E-78
**experimental-after**	0.7	0.0663169557624	0.0664144324101	36948	2.5357295611E-37
**experimental-after**	0.8	0.0276983522213	0.0277054388638	36840	1.05079992823E-07
**experimental-after**	0.9	-0.00456572544097	-0.00456575716684	36732	0.381544258122
**experimental-after**	1	-0.0241649213928	-0.0241696266905	36624	3.73808262448E-06
**experimental-after**	1.1	-0.0344713139481	-0.0344849774508	36516	4.4045044E-11
**experimental-after**	1.2	-0.038935022258	-0.0389547145059	36408	1.06240199247E-13

**experimental-after**	1.3	-0.0540029246198	-0.0540555131981	36300	7.12282879544E-25
**experimental-after**	1.4	-0.0759138227498	-0.076060157209	36192	1.87928661677E-47
**experimental-after**	1.5	-0.0925527821263	-0.0928184183184	36084	1.40944732534E-69

3.2. Study 2 cross-correlation plot data (Figure 4)

CURVE	TIME	CORRELATION	DOF	FISHER_Z	P-VALUE
**strangers**	-1.5	0.00219001390345	0.00219001740468	94674	0.500406981994
**strangers**	-1.4	-0.0180435871852	-0.0180455457243	94962	2.68389688349E-08
**strangers**	-1.3	-0.0351212781706	-0.0351357296154	95250	2.13419844523E-27
**strangers**	-1.2	-0.0411469915945	-0.0411702368592	95538	4.27281705977E-37
**strangers**	-1.1	-0.047118724074	-0.0471536411903	95826	2.94448037184E-48
**strangers**	-1	-0.0440960348854	-0.0441246492729	96114	1.34360654478E-42
**strangers**	-0.9	-0.0361344072015	-0.0361501463766	96402	3.103908323E-29
**strangers**	-0.8	-0.0317829343458	-0.0317936427326	96690	4.77640034756E-23
**strangers**	-0.7	-0.0216037878443	-0.0216071497854	96978	1.7113221952E-11
**strangers**	-0.6	-0.0122779896613	-0.0122786066821	97266	0.000128461208514
**strangers**	-0.5	-0.00655551402737	-0.00655560793701	97554	0.0406036517163
**strangers**	-0.4	0.00039014153417	0.000390141553964	97842	0.902871195994
**strangers**	-0.3	0.0109640088343	0.0109644481919	98130	0.000593229725679
**strangers**	-0.2	0.0208021367797	0.0208051381209	98418	6.71386999656E-11
**strangers**	-0.1	0.0188047240871	0.0188069411183	98706	3.44874923632E-09
**strangers**	0	0.0162071557753	0.0162085750537	98994	3.40094241483E-07
**strangers**	0.1	0.0140301913465	0.0140311120522	98706	1.04220676694E-05
**strangers**	0.2	0.0101850567425	0.010185408948	98418	0.00139670962502
**strangers**	0.3	0.00621828709976	0.00621836724932	98130	0.0514212351785
**strangers**	0.4	-0.00131814741093	-0.00131814817437	97842	0.680110115157
**strangers**	0.5	-0.00580178024767	-0.00580184534623	97554	0.0699665382346
**strangers**	0.6	-0.00649034829685	-0.00649043943364	97266	0.0429489979565
**strangers**	0.7	-0.0101283411756	-0.0101286875298	96978	0.00160936517226
**strangers**	0.8	-0.0089680817048	-0.00896832214018	96690	0.00529198687382
**strangers**	0.9	-0.00564875048636	-0.00564881056834	96402	0.0794509623846
**strangers**	1	-0.00617929273334	-0.00617937138448	96114	0.0553976050458
**strangers**	1.1	-0.00448588473026	-0.00448591482069	95826	0.164939298252

**strangers**	1.2	-0.00281477613509	-0.00281478356891	95538	0.384285266638
**strangers**	1.3	-0.00847609464828	-0.00847629764306	95250	0.00889650650686
**strangers**	1.4	-0.0159444081751	-0.0159457595325	94962	8.93141522665E-07
**strangers**	1.5	-0.016735947236	-0.0167375100332	94674	2.60503182637E-07
**friends**	-1.5	-0.0292086442232	-0.0292169548797	98354	5.05406210447E-20
**friends**	-1.4	-0.0333031309265	-0.0333154512774	98678	1.24548185938E-25
**friends**	-1.3	-0.0381952514977	-0.0382138418341	99002	2.66346649322E-33
**friends**	-1.2	-0.0379502906537	-0.0379685253938	99326	5.34383454464E-33
**friends**	-1.1	-0.0261512524725	-0.0261572164292	99650	1.49191810693E-16
**friends**	-1	-0.00785847038326	-0.00785863215732	99974	0.0129626201337
**friends**	-0.9	0.00854506145752	0.00854526944795	100298	0.00680427092836
**friends**	-0.8	0.0146076529021	0.0146086920459	100622	3.58634118648E-06
**friends**	-0.7	0.0125772051405	0.0125778683831	100946	6.43563890882E-05
**friends**	-0.6	0.00784893369722	0.00784909488302	101270	0.0124961966925
**friends**	-0.5	0.00346700661368	0.00346702050508	101594	0.269128540791
**friends**	-0.4	0.00104663661658	0.00104663699876	101918	0.738277945693
**friends**	-0.3	-0.000570847005016	-0.000570847067023	102242	0.855166751075
**friends**	-0.2	0.00575476071877	0.00575482424735	102566	0.0653237818968
**friends**	-0.1	0.0152455078215	0.0152466891345	102890	1.00529152631E-06
**friends**	0	0.0182153667345	0.0182173817527	103214	4.83695277217E-09
**friends**	0.1	0.0112942764519	0.0112947567238	102890	0.000291251574897
**friends**	0.2	-0.00138221553281	-0.00138221641306	102566	0.658006166992
**friends**	0.3	-0.0122099253817	-0.0122105321971	102242	9.44771466464E-05
**friends**	0.4	-0.0098453113123	-0.00984562943332	101918	0.00167124358676
**friends**	0.5	0.00882250719831	0.00882273611375	101594	0.00492126998319
**friends**	0.6	0.0341755730871	0.0341888877647	101270	1.4370183087E-27
**friends**	0.7	0.0532753726307	0.0533258618541	100946	2.17916486976E-64
**friends**	0.8	0.0637004505513	0.0637868210433	100622	4.93503131877E-91
**friends**	0.9	0.065432227652	0.0655258482638	100298	1.17733375664E-95
**friends**	1	0.0624691538072	0.0625506043787	99974	4.63657581847E-87
**friends**	1.1	0.0593905406459	0.0594605169568	99650	1.32555133313E-78
**friends**	1.2	0.057954934582	0.0580199515097	99326	1.07802777019E-74
**friends**	1.3	0.0542391974858	0.0542924801627	99002	1.9896975829E-65

**friends**	1.4	0.0410271639751	0.0410502066108	98678	4.79492375249E-38
**friends**	1.5	0.0175147762666	0.0175165675837	98354	3.94161071204E-08

3.3. Supplementary plots data

3.3.1. Control condition (Supplementary figure 1)

CURVE	TIME	CORRELATION	DOF	FISHER_Z	P-VALUE
**control-before**	-1.5	-0.0366436975981	-0.036660112062	26803	1.95076002855E-09
**control-before**	-1.4	-0.058814948761	-0.0588829073879	26899	4.57611708022E-22
**control-before**	-1.3	-0.0606254634692	-0.0606999029195	26995	1.99914133431E-23
**control-before**	-1.2	-0.0581161218219	-0.0581816834831	27091	1.00561116278E-21
**control-before**	-1.1	-0.0525571032654	-0.0526055755634	27187	4.17712943846E-18
**control-before**	-1	-0.0389402528597	-0.0389599530499	27283	1.23281534676E-10
**control-before**	-0.9	-0.0251960577815	-0.0252013916462	27379	3.04634810167E-05
**control-before**	-0.8	-0.0129608336262	-0.0129615594335	27475	0.0316776886327
**control-before**	-0.7	0.000304185926495	0.000304185935877	27571	0.959717090905
**control-before**	-0.6	0.0065990576725	0.00659915346596	27667	0.272351409245
**control-before**	-0.5	0.00951701782267	0.00951730516856	27763	0.112785698191
**control-before**	-0.4	0.0140356327507	0.0140365545282	27859	0.019137408664
**control-before**	-0.3	0.0224165273016	0.0224202832076	27955	0.000177813777475
**control-before**	-0.2	0.0332534459151	0.0332657111816	28051	2.52576722832E-08
**control-before**	-0.1	0.0376892364047	0.0377070972135	28147	2.51361271777E-10
**control-before**	0	0.0290525610037	0.0290607390964	28243	1.04035703487E-06
**control-before**	0.1	0.0237245777648	0.023729030439	28147	6.86157004842E-05
**control-before**	0.2	0.0278834513226	0.0278906810345	28051	2.9936944937E-06
**control-before**	0.3	0.0226101166162	0.0226139706932	27955	0.00015620024269
**control-before**	0.4	0.0204186169153	0.0204214552681	27859	0.000653095286855
**control-before**	0.5	0.0189568194607	0.018959090731	27763	0.00158307240569
**control-before**	0.6	-0.00453606995693	-0.0045361010686	27667	0.450544338788
**control-before**	0.7	-0.0303803911519	-0.0303897430427	27571	4.50989788878E-07
**control-before**	0.8	-0.043163516924	-0.0431903527563	27475	8.12360416208E-13
**control-before**	0.9	-0.0513706905279	-0.0514159504036	27379	1.77582674733E-17
**control-before**	1	-0.0505580194465	-0.0506011629762	27283	6.37468372502E-17
**control-before**	1.1	-0.0337899379779	-0.0338028067937	27187	2.49572282636E-08

**control-before**	1.2	-0.0162136655244	-0.016215086514	27091	0.00761008186187
**control-before**	1.3	-0.00447681994683	-0.00447684985521	26995	0.462002543424
**control-before**	1.4	0.00550456976608	0.00550462536377	26899	0.366627323913
**control-before**	1.5	0.00958390353892	0.00958419698612	26803	0.116627323196
**control-after**	-1.5	0.0320467820075	0.0320577594141	58653	8.23760369932E-15
**control-after**	-1.4	0.0256730606084	0.0256787032631	58881	4.63342907845E-10
**control-after**	-1.3	0.0176835984217	0.0176854420449	59109	1.70994553458E-05
**control-after**	-1.2	0.00756212919083	0.00756227334458	59337	0.0654593072027
**control-after**	-1.1	-0.00412258048867	-0.00412260384425	59565	0.314338466409
**control-after**	-1	-0.0197859274635	-0.0197885100211	59793	1.30621053799E-06
**control-after**	-0.9	-0.03395184922	-0.0339649040002	60021	8.71328785626E-17
**control-after**	-0.8	-0.048500070555	-0.0485381525243	60249	1.00040758036E-32
**control-after**	-0.7	-0.0541986642509	-0.0542518274212	60477	1.32496961175E-40
**control-after**	-0.6	-0.0508347579545	-0.0508786145709	60705	4.76326128928E-36
**control-after**	-0.5	-0.042462439045	-0.042487987447	60933	9.80884981175E-26
**control-after**	-0.4	-0.0283478668274	-0.028355463954	61161	2.3406518317E-12
**control-after**	-0.3	-0.00547545967779	-0.00547551439807	61389	0.174890077598
**control-after**	-0.2	0.0214105920142	0.0214138645488	61617	1.06356244503E-07
**control-after**	-0.1	0.0480400580988	0.048077065728	61845	6.03151598529E-33
**control-after**	0	0.0738569055367	0.0739916393791	62073	6.93133164218E-76
**control-after**	0.1	0.0985077126264	0.098828212786	61845	2.21226081281E-133
**control-after**	0.2	0.116955583996	0.117493267017	61617	5.37828096657E-187
**control-after**	0.3	0.127011004111	0.127700663621	61389	1.03624711496E-219
**control-after**	0.4	0.127023653003	0.12771351993	61161	6.02547088756E-219
**control-after**	0.5	0.12194071994	0.12255057095	60933	5.04157776872E-201
**control-after**	0.6	0.108931729277	0.10936568886	60705	6.36491188356E-160
**control-after**	0.7	0.0884923027984	0.0887243853194	60477	1.52621783718E-105
**control-after**	0.8	0.0676238401745	0.0677272048417	60249	4.66336950922E-62
**control-after**	0.9	0.0485313995015	0.0485695553861	60021	1.19566459918E-32
**control-after**	1	0.0300661618018	0.0300752263959	59793	1.92107062723E-13
**control-after**	1.1	0.0138971535351	0.0138980482952	59565	0.000693972061349
**control-after**	1.2	0.00150122160528	0.00150122273303	59337	0.714599651108
**control-after**	1.3	-0.00634197082649	-0.00634205585449	59109	0.123097326348

**control-after**	1.4	-0.0138584306717	-0.0138593179727	58881	0.000770947838963
**control-after**	1.5	-0.0232238243569	-0.0232280009343	58653	1.85029423285E-08

3.3.2. Experimental condition (Supplementary figure 2)

CURVE	TIME	CORRELATION	FISHER_Z	DOF	P-VALUE
**experimental-before**	-1.5	0.0546985921479	0.0547532418485	33695	9.12973369924E-24
**experimental-before**	-1.4	0.0533256473817	0.0533762798398	33827	9.5105815459E-23
**experimental-before**	-1.3	0.0262837882343	0.0262898433535	33959	1.26813419948E-06
**experimental-before**	-1.2	0.00460934380309	0.00460937644695	34091	0.394733988709
**experimental-before**	-1.1	0.00179890511397	0.00179890705442	34223	0.739294255836
**experimental-before**	-1	-0.00296355873864	-0.00296356741468	34355	0.582799423906
**experimental-before**	-0.9	-0.00589478320458	-0.00589485148423	34487	0.273642059043
**experimental-before**	-0.8	0.0163815008625	0.0163829664432	34619	0.0023018254848
**experimental-before**	-0.7	0.0189007221454	0.0189029733089	34751	0.000425372353555
**experimental-before**	-0.6	0.0182465408092	0.0182485661922	34883	0.000653730333789
**experimental-before**	-0.5	0.0328701139301	0.0328819597203	35015	7.60454613962E-10
**experimental-before**	-0.4	0.0173294152845	0.0173311503215	35147	0.00115745070231
**experimental-before**	-0.3	0.00175360854625	0.00175361034379	35279	0.741871918221
**experimental-before**	-0.2	-0.00760677352215	-0.00760692024416	35411	0.152299610928
**experimental-before**	-0.1	-0.0225358666508	-0.0225396828752	35543	2.14369816954E-05
**experimental-before**	0	-0.0120721114883	-0.0120726979862	35675	0.0225916632266
**experimental-before**	0.1	-0.00826044562375	-0.00826063351517	35543	0.119383863232
**experimental-before**	0.2	-0.005453693981	-0.00545374805131	35411	0.304761513694
**experimental-before**	0.3	-0.0190198005012	-0.0190220944879	35279	0.000353098359811
**experimental-before**	0.4	-0.0449570781668	-0.044987403116	35147	3.33965836742E-17
**experimental-before**	0.5	-0.0499427978273	-0.0499843839063	35015	8.50146693782E-21
**experimental-before**	0.6	-0.0247565356009	-0.0247615951067	34883	3.75103070717E-06
**experimental-before**	0.7	-0.000250882860845	-0.000250882866109	34751	0.96269763066
**experimental-before**	0.8	0.0131456572712	0.0131464145759	34619	0.01444326549
**experimental-before**	0.9	0.0296793250245	0.0296880440997	34487	3.52226395392E-08
**experimental-before**	1	0.0366780787351	0.0366945394702	34355	1.03628701681E-11
**experimental-before**	1.1	0.0448416696624	0.0448717614833	34223	1.032097393E-16
**experimental-before**	1.2	0.0601077663702	0.0601803123541	34091	1.10239980726E-28

**experimental-before**	1.3	0.0637768223736	0.0638635044005	33959	5.65589306932E-32
**experimental-before**	1.4	0.0570869268172	0.0571490622152	33827	7.69369269892E-26
**experimental-before**	1.5	0.0455117141064	0.0455431762655	33695	6.27206231277E-17
**experimental-after**	-1.5	-0.013746527037	-0.0137473930152	24394	0.031781549156
**experimental-after**	-1.4	-0.0398589479737	-0.039880076563	24478	4.39207272608E-10
**experimental-after**	-1.3	-0.0472980930142	-0.0473334107715	24562	1.18705502387E-13
**experimental-after**	-1.2	-0.0297951574849	-0.0298039790819	24646	2.88361865421E-06
**experimental-after**	-1.1	-0.0228539634451	-0.0228579435953	24730	0.000324904337395
**experimental-after**	-1	-0.0055538237818	-0.00555388088534	24814	0.381643081238
**experimental-after**	-0.9	0.0338844650248	0.0338974421941	24898	8.85826965209E-08
**experimental-after**	-0.8	0.0847387408561	0.0849424457515	24982	4.27271232387E-41
**experimental-after**	-0.7	0.127410962769	0.128107201133	25066	1.84508354169E-91
**experimental-after**	-0.6	0.141462805173	0.142417938253	25150	5.98931271455E-113
**experimental-after**	-0.5	0.153502105419	0.154725094246	25234	2.14977053828E-133
**experimental-after**	-0.4	0.150833027956	0.151992747606	25318	3.23698772504E-129
**experimental-after**	-0.3	0.136157259434	0.137008141649	25402	1.04767879758E-105
**experimental-after**	-0.2	0.112633847522	0.113113811658	25486	6.84141874123E-73
**experimental-after**	-0.1	0.0728410515762	0.0729702903972	25570	1.84859764173E-31
**experimental-after**	0	0.036638734428	0.0366551422195	25654	4.33161069657E-09
**experimental-after**	0.1	0.0191131161618	0.019115444084	25570	0.00223804214484
**experimental-after**	0.2	0.0204769375131	0.0204798002606	25486	0.00107751458086
**experimental-after**	0.3	0.0331300090638	0.0331421381909	25402	1.27644804621E-07
**experimental-after**	0.4	0.0457011890038	0.0457330460829	25318	3.41645281249E-13
**experimental-after**	0.5	0.0623004347809	0.0623812261552	25234	3.78949692774E-23
**experimental-after**	0.6	0.081851503054	0.0820350340258	25150	1.07718901577E-38
**experimental-after**	0.7	0.069246228612	0.069357227617	25066	4.72684268594E-28
**experimental-after**	0.8	0.0412398721618	0.0412632753034	24982	6.94003023555E-11
**experimental-after**	0.9	0.00800966737194	0.00800983866466	24898	0.206272935895
**experimental-after**	1	-0.0219385710175	-0.0219420917189	24814	0.000547403193847
**experimental-after**	1.1	-0.0311208029135	-0.0311308556337	24730	9.80220295071E-07
**experimental-after**	1.2	-0.0378300171564	-0.0378480789758	24646	2.81931977377E-09
**experimental-after**	1.3	-0.0432091247621	-0.0432360458145	24562	1.23489556895E-11
**experimental-after**	1.4	-0.0380182943693	-0.0380366273674	24478	2.66508708511E-09

**experimental-after**	1.5	-0.039049963443	-0.0390698307153	24394	1.04649616891E-09
